# Integrative morphological, palynological, and phylogenomic evidence supports the recognition of *Hongdaicitrus* as a new genus within *Citrus* sensu lato

**DOI:** 10.3389/fpls.2026.1905317

**Published:** 2026-09-03

**Authors:** Yarong Wang, Ning Kang, Zhiyi Xie, Caiqin Liu, Yue Zhang, Wanyi Zhao, Qiang Fan, Wenbo Liao

**Affiliations:** 1 State Key Laboratory of Biocontrol and Guangdong Provincial Key Laboratory of Plant Stress Biology, School of Life Sciences, Sun Yat-sen University, Guangzhou, China; 2 Guangdong Xiangtoushan National Nature Reserve Administration, Huizhou, China; 3 Guangdong Ecological and Environmental Monitoring Center, Guangzhou, China

**Keywords:** *Citrus* sensu lato, *Hongdaicitrus*, molecular phylogeny, new genus, South China, systematics

## Abstract

Resolving the evolutionary relationships and generic boundaries of *Citrus* sensu lato (s.l.) remains a major challenge in plant systematics, largely because complex reticulate evolution and insufficient sampling of wild lineages have obscured the recognition of independent evolutionary entities. Here, we describe *Hongdaicitrus* gen. nov. and *Hongdaicitrus xiangtoushanensis* sp. nov., a new genus and species within *Citrus* s.l., from Guangdong Province, southern China. The new genus is characterized by imbricate petals that remain semi-closed, campanulate to tubular, and incompletely expanded at anthesis; 19–20 stamens arranged in three whorls, with distinctly unequal filaments connate into five fascicles; a 5-loculed ovary, short terete style, and conical stigma; and absent or highly reduced juice vesicles. Pollen micromorphological observations further support its distinctiveness, showing four relatively shallow germination sulci and densely perforate to finely foveolate exine ornamentation with small and relatively uniform lumina. Phylogenomic analyses based on 83 plastid protein-coding sequences and 5, 873 single-copy nuclear genes consistently recovered *Hongdaicitrus* as a deeply divergent lineage sister to *Fortunella* and clearly separated from *Citrus* s.s. Genome-wide resequencing analyses further showed that *Hongdaicitrus* forms a distinct genetic cluster and is clearly differentiated from *Fortunella*, *Citrus* s.s., *Papeda*, *Poncirus*, and other citrus relatives. Ecologically, *Hongdaicitrus* is endemic to rock crevices of exposed granite outcrops with extremely barren soils. Based on the IUCN Red List criteria, *Hongdaicitrus xiangtoushanensis* is provisionally assessed as Critically Endangered (CR). Our integrative evidence supports the recognition of *Hongdaicitrus* as a distinct genus, provides new insights into generic delimitation, systematics, and biogeographic diversification among *Citrus* s.l.

## 1 Introduction

Generic delimitation of *Citrus* and its allied lineages remains among the most complex and contentious systematic challenges in angiosperm systematics (Tanaka, 1969). Historically, classifications relied primarily on traditional morphology focusing on fruit, leaf, and floral characters (Swingle and Reece, 1967; Talon et al., 2020; Goldschmidt and Bar-Joseph, 2023). However, extensive phenotypic continuity across the clade, compounded by complex interspecific hybridization in cultivated accessions, has fueled protracted debates regarding generic circumscriptions and culminated in two classical taxonomic frameworks (Swingle, 1967; Zhang et al., 2008; Bayer et al., 2009; Mabberley, 2022). The first, exemplified by Swingle’s system, emphasizes hybridization and morphological continuity. Swingle’s system applies a comparatively conservative species circumscription while recognizing six genera within the true citrus fruit trees, including *Citrus*, *Fortunella*, *Poncirus*, *Microcitrus*, *Eremocitrus*, and *Clymenia*, with *Papeda* treated as a subgenus of *Citrus* (Swingle, 1943; Swingle and Reece, 1967). Conversely, the alternative classification system proposed by Tanaka prioritizes fine-scale morphological divergence and biogeographic structure, advocating further subdivision of *Citrus* sensu lato (s.l.) at both the generic and subgeneric levels (e.g., *Pleurocitrus*, *Sinocitrus*) and the recognition of putatively primitive and derived lineages within the clade (Tanaka, 1954b).

Modern broad treatments, including those adopted in several floristic accounts and taxonomic databases, incorporate some or all of the traditionally segregated genera into *Citrus* (Mabberley, 1997, 2004, 2022; Zhang et al., 2008). For example, Plants of the World Online accepts *C. japonica* rather than *Fortunella japonica* and *C. trifoliata* rather than *Poncirus trifoliata*, and comparable combinations are used in NCBI Taxonomy records (Schoch et al., 2020; POWO, 2026). The widespread use of this broad circumscription has a historical and practical basis and facilitates nomenclatural continuity and communication in horticulture, germplasm management, and biological databases. At the same time, phylogenetic and genomic studies consistently recover several deeply differentiated and morphologically diagnosable lineages within the true citrus group, particularly *Fortunella* and *Poncirus* (Abkenar et al., 2004; Carbonell-Caballero et al., 2015; Zhu et al., 2022; Huang et al., 2023). The monophyly of the inclusive true citrus group establishes the common ancestry of these lineages but does not, by itself, determine the appropriate taxonomic rank or circumscription of its constituent clades.

In the present study, we therefore evaluate the Xiangtoushan lineage under a segregate, phylogenetically informed generic framework, in which *Fortunella*, *Poncirus*, and *Papeda* are treated as genus-level units and *Citrus* s.s. is restricted to the core *Citrus* lineage. The term *Citrus* s.l. is used only as an informal collective designation for the entire true citrus group, including *Citrus* s.s. and its traditionally segregated allied genera. Under this framework, recognition of an additional genus cannot be considered an isolated taxonomic act, but requires other independently differentiated lineages to be evaluated according to the same integrative criteria and ultimately has implications for the recircumscription of *Citrus* s.s. This framework provides an explicit basis for evaluating whether the Xiangtoushan lineage merits recognition at generic rank, while a comprehensive segregate classification of the entire true citrus group will require broader systematic and nomenclatural reassessment.

In reticulate groups such as *Citrus* s.l., hybridization and backcrossing may generate mosaic or intermediate character combinations through introgression, whereas apomixis can perpetuate such genotypes (Koltunow, 1993; Wang et al., 2017; Wu et al., 2018). Morphology alone may be insufficient for generic delimitation because some characters are influenced by environmental conditions, and morphological similarity does not necessarily reflect common ancestry. Consequently, morphology may either obscure genetically distinct lineages or overemphasize variation within a single lineage (Iwata et al., 2002; Duminil and Di Michele, 2009). Morphology therefore remains indispensable for taxonomic diagnosis, but generic delimitation is more reliable when stable combinations of diagnostic characters are evaluated together with phylogenetic and genomic evidence (Humphreys and Linder, 2009). Molecular systematic studies have also revealed frequent incongruence between plastid and nuclear signals (Bayer et al., 2009; Carbonell-Caballero et al., 2015), further complicating the establishment of consistent genus-level boundaries in a phylogenetic context (Tanaka, 1954a; Batygina and Vinogradova, 2007; Koltunow, 1993; Nakano et al., 2012; Wang et al., 2017, 2022). Consequently, both research and applied practice have widely adopted *Citrus* s.l. as an operational taxonomic framework encompassing multiple closely related lineages (Bayer et al., 2009; Mabberley, 1997, 2022; Mabberley, 2004; Scora, 1975; Wu et al., 2018; Zhang et al., 2008). Nevertheless, the phylogenetic coherence of this broad grouping and the criteria for genus delimitation within it remain subjects of ongoing debate (Fang et al., 2018; Huang et al., 2023). This ambiguity in the taxonomic framework, in turn, can complicate the documentation, conservation, and utilization of *Citrus* germplasm resources. A concrete nomenclatural example is *Citrus reticulata*: Mabberley and Xu (2022) pointed out that Blanco’s name was based on plants cultivated in the Philippines and associated with the *C. × aurantium* complex, leading major databases such as POWO to treat *C. reticulata* as a synonym of *C. × aurantium*. To remove this uncertainty and maintain the widespread modern use of the name for mandarin, they proposed to conserve C. reticulata with pure mandarin material of Mangshan origin as its conserved type (Mabberley, 1997; Mabberley and Xu, 2022).

Mabberley (2004) emphasized that the critical gap is confirming truly wild citrus populations in Asia (Mabberley, 2004). Voucher-documented wild germplasm is particularly important because phylogenetic patterns inferred predominantly from cultivated, admixed, or clonally maintained accessions may not accurately represent the original differentiation of wild lineages. With accumulating evidence from morphology, reproductive biology, and biogeography, certain lineages exhibiting marked differentiation in form, reproductive traits, and distribution continue to be segregated from *Citrus* and accorded generic status in various classification systems (Wu et al., 2018). *Fortunella*, for example, constitutes a stable and deeply divergent monophyletic clade across chloroplast, nuclear, and whole-genome phylogenetic frameworks, offering strong and consistent support for its evolutionary independence (Zhang et al., 2006; Zhu et al., 2022). Such lineages further display diagnostically informative and relatively consistent differences in flowering phenology, fruit structure, leaf morphology, and ecological adaptation (Bayer et al., 2009; Traband et al., 2023). Accordingly, as evidence from voucher-documented wild resources continues to accumulate (Huang et al., 2023; Wu et al., 2021), the purpose of a segregate framework is not to maximize the number of genera, but to provide explicit taxonomic recognition for lineages that are consistently supported as evolutionarily independent and morphologically diagnosable, while maintaining an internally coherent classification (Humphreys and Linder, 2009; Bouman et al., 2021, 2022).

In recent years, we have conducted several surveys of the *Citrus* s.l. resources in southern China. Our field studies in the Xiangtoushan National Nature Reserve, Guangdong province have revealed a citrus-related lineage exhibiting a distinctive combination of vegetative and reproductive traits. This suite of characters does not closely match the diagnoses of the traditional genus-level lineages compared here within the operational framework of *Citrus* s.l. Although the lineage shares some features with certain groups traditionally included in *Citrus* s.l., its overall morphology, along with its phylogenetic position, supports its status as an evolutionarily distinct entity. We therefore suspect that it represents a previously overlooked independent lineage within the citrus complex.

To clarify the phylogenetic position and taxonomic significance of the Xiangtoushan lineage, we integrated plastid and nuclear phylogenomics, genome-wide resequencing, comparative morphology, pollen micromorphology, and ecological observations. Specifically, this study aimed to: (1) reconstruct the phylogenetic placement of the Xiangtoushan lineage using plastid and nuclear genomic datasets; (2) assess its genome-wide differentiation from *Fortunella*, *Citrus*, *Papeda*, and other citrus relatives; (3) evaluate its diagnostic morphological and palynological characters; and (4) determine whether this lineage represents an evolutionarily distinct entity that merits recognition as a separate genus under the segregate, phylogenetically informed framework adopted here.

## 2 Materials and methods

### 2.1 Plant material sampling

From late January to December in 2024 and 2025, we conducted field investigations in Xiangtoushan National Nature Reserve, Guangdong, southern China, covering both flowering and fruiting periods of several unidentified Rutaceae plants. Voucher specimens were collected and deposited in the Herbarium of Sun Yat-sen University (SYS, herbarium acronyms following Thiers, 2026). To minimize potential impacts on natural populations, field sampling followed the conservative collection strategy proposed by Pavlovic et al. (1992), in which the number of collected specimens was kept proportional to local population size and only the minimum material necessary for taxonomic and molecular analyses was taken (Pavlovic et al., 1992). Freshly opened flowers, one from each plant, were collected and immediately preserved in 75% ethanol for further processing in the laboratory. Seeds were collected in paper bags, appropriately dried, and stored in silica gel. Leaf material from several individuals was collected, rapidly desiccated in silica gel, and stored for subsequent DNA extraction.

### 2.2 Morphological observations

The flowering and fruiting plants of the new genus were examined in the field from late January 2024 to 2025 and compared with herbarium specimens deposited in SYS (herbarium acronyms following Thiers, 2026). Morphological comparisons between the Xiangtoushan lineage and other *Citrus* s.l. taxa were first conducted by consulting relevant taxonomic literature, including the *Flora of China* (Zhang et al., 2008; Wang et al., 2022a; Huang et al., 2023). Because the Xiangtoushan plants showed the greatest morphological similarity to *Fortunella*, we further compared them in detail with representative species of *Fortunella*. Morphological characters distinguishing the putative new genus from *Fortunella*, *Papeda* and *Citrus* s.s. were assessed through examination of herbarium specimens deposited in SYS, PE, IBK, KUN, IBSC, HIB, and NAS, supplemented by comparisons with representative type-associated materials, including *Fortunella hindsii* (R.B. Hinds s.n., K000717994, K) and *Papeda ichangensis* (Cavalerie 2936, P5240963, P), with herbarium acronyms following Thiers (2026).

### 2.3 Pollen scanning electron microscopy

Pollen micromorphology was examined using scanning electron microscopy (SEM). Dry mature pollen grains were obtained from flower buds or freshly opened flowers of the Xiangtoushan lineage. Mature anthers were gently crushed onto aluminum stubs with double-sided adhesive tape, and the pollen grains were sputter-coated with gold before observation.

The prepared samples were examined using a scanning electron microscope at an accelerating voltage of 20 kV. Representative images were taken in ensemble, polar, and equatorial views. Aperture morphology and exine ornamentation were documented at higher magnification. Pollen terminology and descriptive criteria followed standard palynological references (Moore and Webb, 1978; Wei, 2003) and previous studies on *Fortunella* pollen morphology (Wang et al., 2022b).

### 2.4 Phylogenetic analyses

Fresh leaf material from each sampled individual was collected and stored in silica gel for subsequent molecular experiments. Whole genomic DNA for each sample was extracted using a modified CTAB method (Doyle, 1991). Short-insert genomic DNA libraries with an average insert size of approximately 400 bp were constructed following the standard Illumina library preparation workflow according to the manufacturer’s instructions. Paired-end sequencing with a read length of 150 bp was performed on an Illumina NovaSeq 6000 platform. Chloroplast genomes were assembled using GetOrganelle v1.7.7.0 (Jin et al., 2020) and annotated with cpGAVAS (Liu et al., 2012). Voucher specimens were deposited in the Herbarium of Sun Yat-sen University (SYS). The plastid dataset comprised 48 accessions representing 24 species-level taxa, including the three Xiangtoushan accessions generated in this study and 45 publicly available accessions. The nuclear dataset comprised 19 accessions representing 17 species-level taxa and included both newly generated and publicly available genomic data (**Supplementary Tables 1, 2**). The raw whole-genome resequencing data generated in this study have been submitted to the National Genomics Data Center (NGDC; https://ngdc.cncb.ac.cn/) under BioProject accession PRJCA059069, with individual sample accessions and run accessions provided in **Supplementary Table 2**.

Chloroplast CDS sequences were aligned separately for each gene using MAFFT (Katoh et al., 2002). The resulting alignments were then trimmed with Gblocks (Castresana, 2000) under codon mode to remove unreliable regions, and all filtered gene alignments were concatenated into a chloroplast CDS supermatrix. The best-fitting nucleotide substitution model for the concatenated matrix was evaluated using ModelTest-NG v0.2.0 (Darriba et al., 2020). Maximum likelihood (ML) phylogenies were inferred with RAxML-NG (Kozlov et al., 2019), under the TVM+I+G4 substitution model. Tree searches were performed using 25 parsimony-based and 25 random starting trees, and branch support was assessed with 30, 000 bootstrap replicates. Two species of *Atalantia*, *A. buxifolia* and *A. kwangtungensis*, were included as outgroups because *Atalantia* lies outside the core *Citrus* s.l. group. Bayesian inference (BI) was performed in MrBayes v3.2.7a (Ronquist et al., 2012). The GTR+I+G model was applied with four gamma rate categories. Two independent MCMC runs were conducted, each with four chains for 2, 000, 000 generations, sampling every 1, 000 generations. Convergence was assessed using the average standard deviation of split frequencies, with a stop criterion of 0.01. The first 25% of sampled trees were discarded as burn-in, and posterior probabilities were calculated from the remaining trees.

We retrieved predicted protein sequences (.pep) for seven published genome assemblies from the *Citrus* Pan Genome to Breeding Database (Liu et al., 2022), including *Atalantia buxifoliata* (Wang et al., 2017), *C. mangshanensis* (Liu et al., 2025), *C. mangshanyeju* (Liu et al., 2025), *C. maxima* (Huang et al., 2023), *C. medica* (Wang et al., 2017), *C. trifoliata* (Huang et al., 2021), and *Fortunella hindsii* (Wu et al., 2025). Using these reference proteomes, we inferred orthogroups and identified a set of single-copy orthologues with OrthoFinder (Emms and Kelly, 2015, 2019). The resulting single-copy nuclear gene reference set was then used as the target file for HybPiper to recover nuclear genes from resequencing reads of additional taxa, generating coding sequences (CDS) and the corresponding protein sequences for each single-copy locus for downstream phylogenetic analyses. For each locus, protein sequences were aligned with MAFFT (Katoh et al., 2002). Protein alignments were back translated into codon (CDS) alignments using PAL2NAL, with the -nomismatch option to reduce errors caused by inconsistent translations (Suyama et al., 2006); loci that failed back translation (yielding empty outputs) were recorded and excluded. Codon alignments were then trimmed with Gblocks in codon mode to remove unreliable or ambiguously aligned regions (Castresana, 2000).

Gene recovery and missing-data statistics were calculated from the trimmed alignments of 5, 873 single-copy nuclear orthologues. A locus was considered recovered for a sample when its alignment contained at least one unambiguous nucleotide (A, C, G, or T), whereas gaps, Ns, and other ambiguous characters were treated as missing data. Per-sample gene recovery was calculated as the proportion of recovered loci among all 5, 873 orthologues, and taxon occupancy for each locus was calculated as the proportion of the 19 accessions containing recoverable sequence data. The resulting concatenated nuclear alignment was 9, 264, 441 bp long and contained 2.08% missing data. Mean locus occupancy was 99.99%; 5, 865 loci had complete occupancy across all 19 accessions, whereas eight loci contained an entirely missing sequence for at least one accession (**Supplementary Table 7**).

The curated single-copy loci were concatenated into a nuclear supermatrix with a consistent sample order, and the best-fitting nucleotide substitution model for the concatenated matrix was selected using ModelTest-NG v0.2.0 (Darriba et al., 2020). A ML species tree was inferred using RAxML-NG under the GTR+I+G4 model. Tree searches were performed using 25 parsimony-based and 25 random starting trees, and nodal support was assessed with 1, 000 Felsenstein bootstrap replicates. The tree was rooted with *Atalantia buxifoliata* as the outgroup (Kozlov et al., 2019).

To account for variation among individual nuclear loci, separate ML gene trees were reconstructed for the 5, 865 loci with complete taxon occupancy using IQ-TREE v2.2.2.6. The best-fitting substitution model for each locus was selected using ModelFinder with the -m MFP option. The resulting gene trees were analyzed under the multispecies coalescent in ASTRAL-III v5.7.8 (Zhang et al., 2018). The three Xiangtoushan accessions were assigned to *Hongdaicitrus xiangtoushanensis* using a species-mapping file following the multi-individual implementation of ASTRAL (Rabiee et al., 2019). Local posterior probabilities and quartet frequencies (q1, q2, and q3) were calculated for each internal branch to quantify support for the ASTRAL-inferred topology and the two alternative quartet resolutions (Sayyari and Mirarab, 2016).

Gene concordance factors (gCF) were subsequently calculated in IQ-TREE using the concatenated nuclear ML topology as the reference tree and the 5, 865 individual gene trees. Likelihood-based site concordance factors (sCF) were estimated from the corresponding concatenated alignment using 1, 000 quartet samples. The gCF represents the percentage of decisive gene trees supporting a focal branch, whereas the likelihood-based sCF summarizes the proportion of decisive sites supporting that branch (Minh et al., 2020; Mo et al., 2023). Concordance-vector components for the focal *Hongdaicitrus–Fortunella* branch were extracted for comparison with the ASTRAL quartet frequencies.

### 2.5 Population genomic analyses based on whole-genome resequencing data

To further evaluate the genomic distinctiveness of the Xiangtoushan lineage, newly generated whole-genome resequencing data from the three XTS individuals and representative accessions of *Citrus*, *Papeda*, and *Poncirus* were analyzed together with publicly available resequencing data from 11 *Fortunella hindsii* accessions retrieved from NCBI. These accessions were designated as wild in their source studies and associated database metadata on the basis of their collection from natural populations and population-genomic assignments (Zhu et al., 2022; Wang et al., 2023). Their field origins were not independently verified in the present study. Detailed sample information is provided in **Supplementary Table 3**.

Clean reads were mapped to the *Fortunella hindsii* (Zhu et al., 2019) reference genome using BWA-MEM v0.7.17 (Li, 2013). Alignments were sorted with SAMtools (Li et al., 2009), PCR duplicates were marked with Picard, and variant calling was performed following the GATK best practices workflow (McKenna et al., 2010). Genomic VCF (gVCF) files were generated separately for each sample and subsequently subjected to joint genotyping to produce a combined variant dataset.

SNP filtering was performed using PLINK v1.90 (Purcell et al., 2007). Sites with more than 10% missing genotypes were removed, variants with minor allele frequency lower than 0.01 were excluded, and only strictly biallelic SNPs were retained. Non-standard chromosome names were allowed, missing variant IDs were replaced with chromosome-position-based IDs, and the original allele order was preserved. To reduce linkage disequilibrium, SNPs were pruned using a sliding window of 50 SNPs, a step size of 5 SNPs, and an r^2^ threshold of 0.25. The resulting LD-pruned SNP dataset was used for PCA and pairwise IBS distance analyses.

Principal component analysis was performed with PLINK v1.90, and the results were visualized in R. Pairwise genomic distances were calculated as one minus identity by state (1 − IBS) using PLINK v1.90. The resulting distance matrix was used to generate a heatmap and to summarize within and between group genetic distances.

To test for asymmetric allele sharing and possible historical introgression, D-statistics were calculated using the Dtrios module of Dsuite (Malinsky et al., 2021). Unlike the PCA and IBS analyses, this analysis used the SNP dataset before LD pruning because linkage was accounted for through block jackknifing. The dataset was restricted to 29 individuals assigned to five groups: *Hongdaicitrus* (three individuals), *Fortunella* (11), *Papeda* (three), *Citrus reticulata* (10), and *Poncirus* (two). After normalization and restriction to biallelic SNPs, 534, 534 sites were retained. *Poncirus* was specified as the outgroup, and all four possible trios among the remaining groups were evaluated. D-statistics, f4-ratios, Z-scores, and P-values were calculated using 100 block-jackknife replicates. To account for the four trio tests, P-values were additionally adjusted using the Benjamini–Hochberg procedure. Full results are provided in **Supplementary Table 6**.

## 3 Results

### 3.1 Phylogenetic position of the Xiangtoushan lineage

**Figure 1:**
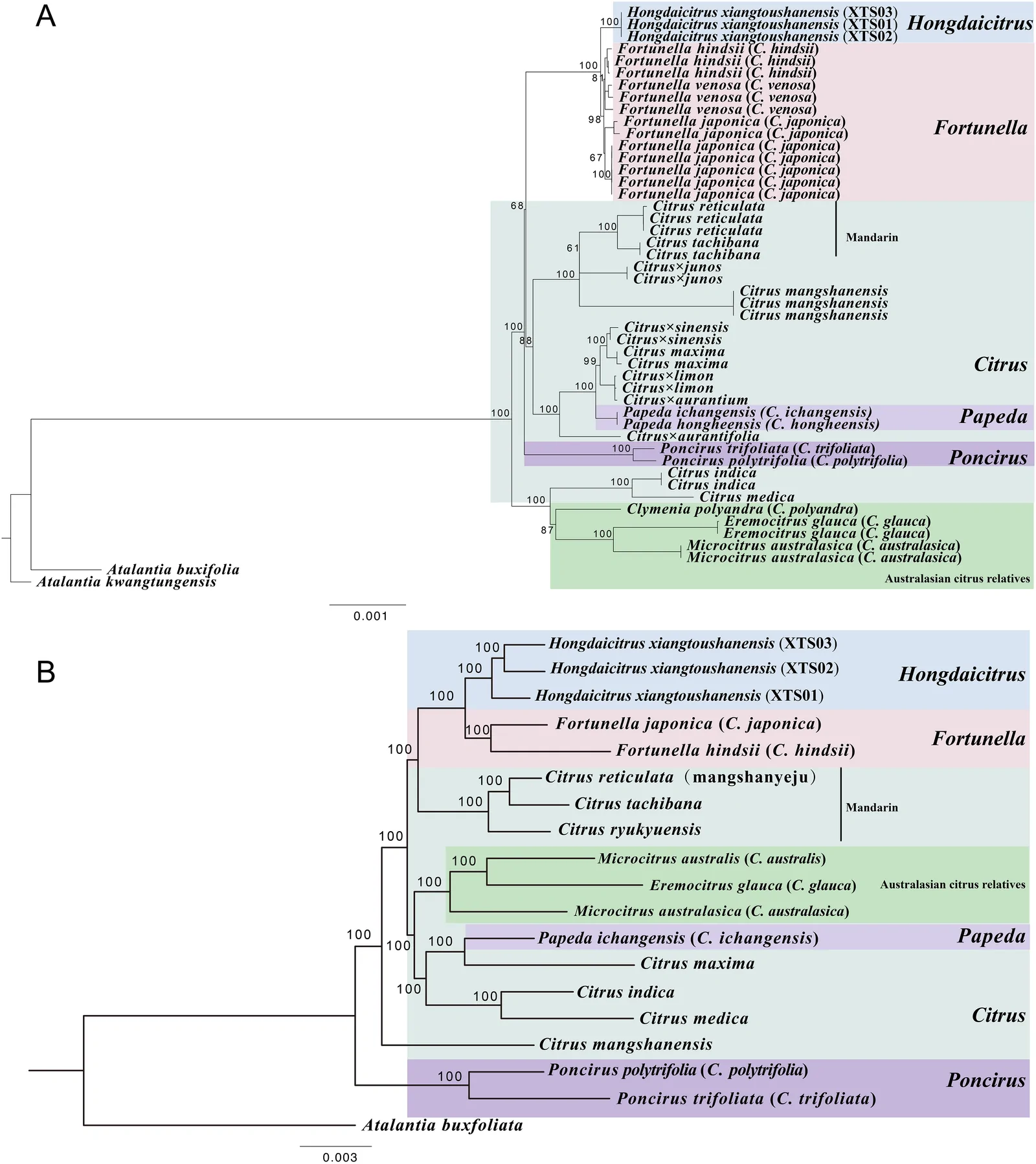
Maximum-likelihood phylogenies of *Citrus* s.l. inferred from plastid and nuclear datasets. **(A)** Phylogeny inferred from a concatenated alignment of 83 plastid protein-coding sequences (CDS), with expanded representation of wild and cultivated *Fortunella* taxa and the traditionally segregated Australasian citrus relatives *Clymenia*, *Eremocitrus*, and *Microcitrus*. **(B)** Phylogeny inferred from 5, 873 single-copy nuclear orthologues. Both trees were reconstructed using RAxML-NG, with bootstrap support values shown at the nodes. Traditional genus-level lineages used for comparison under the segregate framework adopted in this study are indicated by coloured background boxes. The Australian and New Guinean taxa are additionally grouped as “Australasian citrus relatives” to indicate their geographic affinity; this label does not represent a single genus. The bracket labelled “Mandarin” identifies the sampled core mandarin lineage and does not denote a separate genus. Names in parentheses indicate the corresponding combinations under a broadly circumscribed *Citrus* treatment. In both datasets, the three accessions of *Hongdaicitrus xiangtoushanensis* (XTS01–XTS03) form a strongly supported monophyletic clade sister to *Fortunella* rather than being nested within it. *Atalantia buxifolia* and *A. kwangtungensis* were used as outgroups in the plastid analysis, whereas *A. buxifolia* was used as the outgroup in the nuclear analysis. Scale bars indicate substitutions per site.

ML phylogenetic inference based on a concatenated alignment of 83 plastid protein-coding sequences (CDS) recovered the Xiangtoushan lineage as a strongly supported and distinct clade (**Figure 1A**). The plastid dataset included multiple accessions of *Fortunella hindsii*, *F. venosa*, and *F. japonica*, together with representatives of the Australasian genera *Clymenia*, *Eremocitrus*, and *Microcitrus*. The three accessions from Xiangtoushan (XTS01–XTS03) formed a well-supported monophyletic group with maximal bootstrap support (BS = 100) and were resolved as sister to *Fortunella*. This relationship remained stable under the expanded taxon sampling, and the Xiangtoushan lineage was clearly separated from *Citrus* s.s., *Papeda*, *Poncirus*, and the sampled Australasian lineages. BI yielded a highly congruent topology (**Supplementary Figure 1**), likewise supporting the monophyly of the Xiangtoushan lineage with maximal posterior probability (PP = 1.00).

To further validate this phylogenetic placement, we reconstructed an ML tree using 5, 873 single-copy nuclear orthologues (**Figure 1B**). The concatenated nuclear matrix comprised 9, 264, 441 bp, with 2.08% missing data. Per-sample gene recovery ranged from 99.86% to 100%, and 5, 865 loci had complete taxon occupancy (**Supplementary Table 7**). The nuclear dataset also included wild and cultivated representatives of *Fortunella* and representatives of the Australian lineages *Microcitrus* and *Eremocitrus*. Although the deeper positions of several other lineages differed between the plastid and nuclear phylogenies, the sister-group relationship between the Xiangtoushan lineage and *Fortunella* was consistently recovered with maximal support.

**Figure 2:**
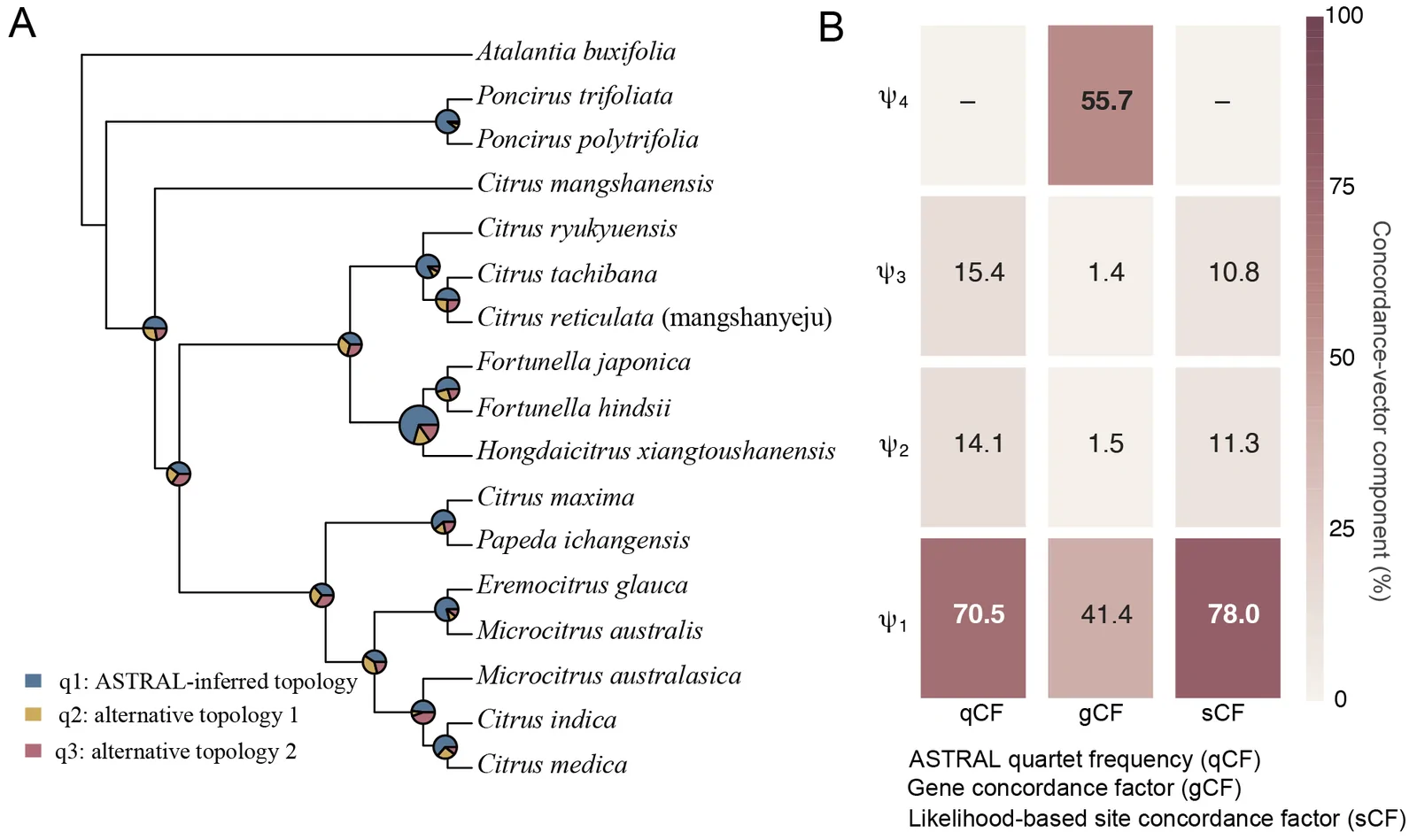
Gene-tree concordance surrounding the phylogenetic placement of *Hongdaicitrus*. **(A)** Multispecies-coalescent species tree inferred with ASTRAL from 5, 865 single-copy nuclear gene trees. Pie charts show local quartet frequencies supporting the ASTRAL-inferred topology (q1, blue) and the two alternative quartet topologies (q2, ochre; q3, rose). The enlarged pie chart denotes the focal branch supporting the sister relationship between *Hongdaicitrus* and *Fortunella*. Branch lengths are not proportional to evolutionary distance. **(B)** Concordance-vector components for the focal *Hongdaicitrus–Fortunella* branch estimated from ASTRAL quartet frequencies, gene concordance factors (gCF), and likelihood-based site concordance factors (sCF). ψ1 represents support for the focal topology, whereas ψ2 and ψ3 represent the two alternative quartet resolutions. For the gene-tree analysis, ψ4 corresponds to the gene discordance factor due to other relationships among groups surrounding the focal branch (gDFP). ψ4 is not applicable to the ASTRAL quartet-frequency or site-concordance analyses. Detailed ASTRAL local quartet statistics are provided in **Supplementary Table 5**.

To assess concordance across individual nuclear loci, we inferred a multispecies-coalescent species tree from 5, 865 single-copy gene trees using ASTRAL (**Figure 2A**). The ASTRAL analysis again recovered *Hongdaicitrus* as sister to *Fortunella*, with a local posterior probability of 1.00. At the focal *Hongdaicitrus–Fortunella* branch, the ASTRAL-inferred topology was supported by 70.55% of informative quartets (q1), whereas the two alternative quartet resolutions accounted for 14.10% (q2) and 15.36% (q3), respectively (**Figure 2A**; **Supplementary Table 5**). Gene concordance analysis recovered a gCF of 41.38% for this branch. Although 55.74% of gene trees supported other discordant resolutions (gDFP), the two principal alternative resolutions individually received little support (gDF1 = 1.48%; gDF2 = 1.40%). Site concordance was substantially higher (sCF = 77.95%), whereas the two alternative site resolutions received 11.26% (sDF1) and 10.79% (sDF2) support (**Figure 2B**). Thus, despite gene-tree heterogeneity, the ASTRAL quartet frequencies and site-level concordance consistently identified the *Hongdaicitrus–Fortunella* sister relationship as the predominant nuclear phylogenetic signal.

Taken together, the plastid and nuclear datasets consistently recover the Xiangtoushan lineage as a monophyletic lineage sister to, rather than nested within, *Fortunella*. Within the sampled framework of *Citrus* s.l., the lineage is also not nested within any other traditional genus-level lineage represented in the present analyses. These results support the evolutionary independence of the Xiangtoushan lineage and, when evaluated together with its stable diagnostic morphological characters, provide molecular phylogenetic evidence for its recognition as the new genus *Hongdaicitrus*.

### 3.2 Genome-wide differentiation of the Xiangtoushan lineage among citrus relatives

**Figure 3:**
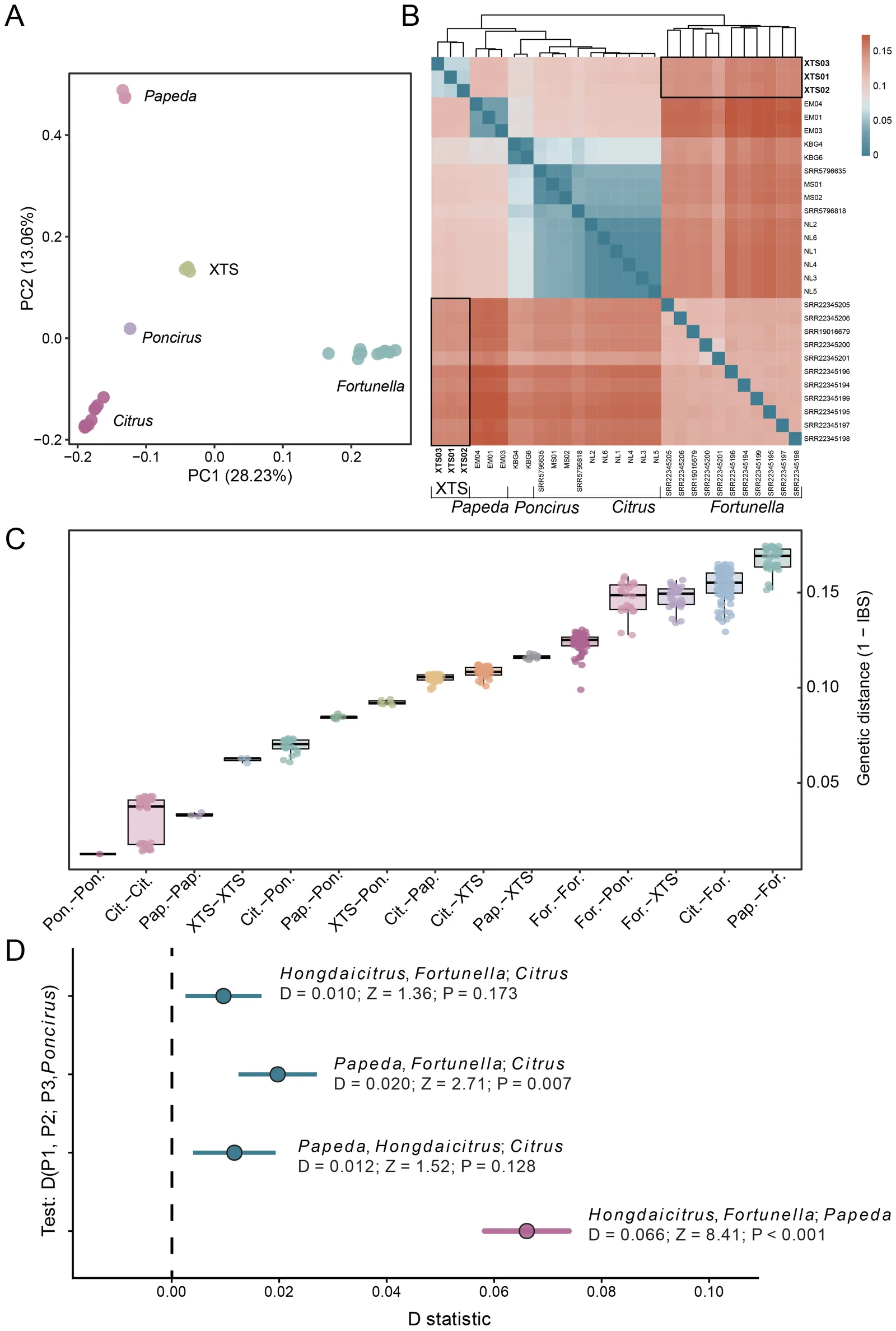
Genome-wide evidence for the independent status of the Xiangtoushan lineage. **(A)** Principal component analysis (PCA) based on genome-wide SNPs separates the Xiangtoushan lineage (*Hongdaicitrus*; XTS) from the sampled *Citrus reticulata*, *Fortunella*, *Poncirus*, and *Papeda* groups. **(B)** Pairwise genetic-distance heatmap showing that XTS forms a cohesive cluster distinct from the other sampled groups. The boxed regions highlight pairwise comparisons between XTS and *Fortunella*. **(C)** Distributions of within- and between-group pairwise genetic distances (1 − IBS). Points represent individual pairwise comparisons, and boxplots show the median and interquartile range. Group abbreviations are as follows: Cit., *C. reticulata*; For., *Fortunella*; Pap., *Papeda*; and Pon., *Poncirus*. **(D)** D-statistics testing asymmetric allele sharing among the sampled lineages, with *Poncirus* used as the outgroup. Points indicate D estimates, and horizontal bars represent ± 1 block-jackknife standard error based on 100 blocks. D-statistics were calculated from 534, 534 biallelic SNPs. The significant positive result for *D*(*Hongdaicitrus*, *Fortunella*; *Papeda*, *Poncirus*) indicates greater allele sharing between *Fortunella* and *Papeda* than between *Hongdaicitrus* and *Papeda*. Exact statistics are provided in **Supplementary Table 6**.

To further evaluate the genomic distinctiveness of the Xiangtoushan lineage, we analyzed whole-genome resequencing data from the three XTS individuals together with representative accessions of *Citrus*, *Papeda*, *Poncirus*, and 11 publicly available *F. hindsii* accessions designated as wild in their source studies (Zhu et al., 2022; Wang et al., 2023; **Supplementary Table 3**). Principal component analysis based on LD-pruned genome-wide SNPs showed that the three XTS individuals formed a compact cluster that was clearly separated from the sampled *Fortunella* accessions (**Figure 3A**). The XTS cluster was also distinct from *Citrus*, *Papeda*, and *Poncirus*, indicating that the Xiangtoushan lineage does not fall within the genomic variation of any sampled reference lineage.

Pairwise genomic distances, calculated as one minus identity by state (1 − IBS), provided a quantitative assessment of this differentiation. In the distance heatmap, XTS01, XTS02, and XTS03 clustered together and were separated from the sampled Fortunella accessions, as well as from Citrus, Papeda, and Poncirus (**Figure 3B**). The mean within-XTS distance was 0.0621 (range = 0.0603–0.0629), consistent with the genetic cohesion of the three Xiangtoushan individuals. In comparison, the mean XTS–*Fortunella* distance was 0.1476 (median = 0.1494; range = 0.1341–0.1566), exceeding the mean distance within *Fortunella* (0.1235; median = 0.1252; range = 0.0989–0.1305). Notably, all 33 XTS–*Fortunella* comparisons exceeded the maximum distance observed among the 55 within-*Fortunella* comparisons. The mean XTS–*Fortunella* distance was also nearly identical to that between *Fortunella* and *Poncirus* (0.1467), although lower than that between *Fortunella* and *Papeda* (0.1673; **Figure 3C**; **Supplementary Table 4**).

To test whether the morphological resemblance between *Hongdaicitrus* and *Papeda* might reflect historical introgression, we calculated D-statistics from 534, 534 biallelic SNPs using *Poncirus* as the outgroup (**Figure 3D**; **Supplementary Table 6**). The focal test, *D*(*Hongdaicitrus*, *Fortunella*; *Papeda*, *Poncirus*), produced a significantly positive value (*D* = 0.0661, *Z* = 8.41, *P* = 2.3 × 10^−16^; BH-adjusted *P* = 9.2 × 10^−16^). Given the P1–P2–P3 ordering, this result indicates greater allele sharing between *Fortunella* and *Papeda* than between *Hongdaicitrus* and *Papeda*. A weaker but significant asymmetry was detected between *Fortunella* and *C. reticulata* relative to *Papeda* (*D* = 0.0197, *Z* = 2.71, *P* = 0.0068; BH-adjusted *P* = 0.0136). By contrast, neither the *Hongdaicitrus–Fortunella–Citrus* comparison (*D* = 0.0096, *Z* = 1.36, *P* = 0.173) nor the *Papeda–Hongdaicitrus–C. reticulata* comparison (*D* = 0.0117, *Z* = 1.52, *P* = 0.128) was significant. Thus, within the sampled lineages, the D-statistics did not detect preferential allele sharing between *Hongdaicitrus* and *Papeda*.

Together, the genome-wide PCA and pairwise 1-IBS distance analyses indicate that the Xiangtoushan lineage is genetically cohesive and does not represent an intraspecific variant of the sampled *Fortunella hindsii* accessions. The D-statistics further provide no evidence that the morphological resemblance between *Hongdaicitrus* and *Papeda* is associated with preferential historical allele sharing between these lineages. Collectively, these analyses provide quantitative genome-wide evidence for the differentiation of the Xiangtoushan lineage, complementing the plastid and nuclear phylogenomic results.

### 3.3 Morphological distinctiveness and diagnostic characters

**Figure 4:**
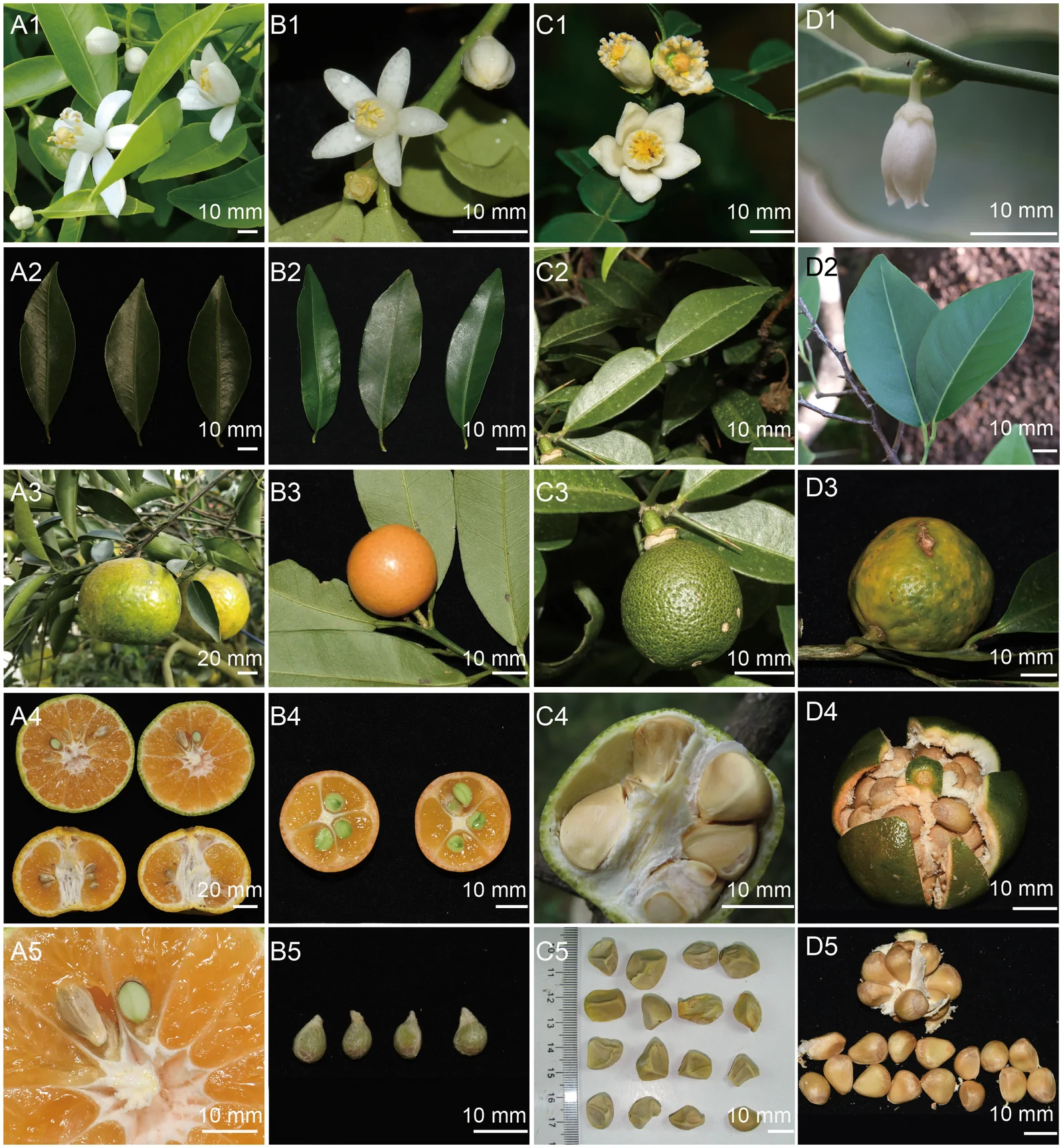
Morphological comparison of *Hongdaicitrus* with selected allied genera. **(A)** *Citrus reticulata* ‘Chachiensis’; **(B)** *Fortunella japonica*; **(C)** *Papeda ichangensis*; **(D)** *Hongdaicitrus xiangtoushanensis.* Rows 1–5 show flowers, leaves, fruits, fruit sections, and seeds, respectively. Scale bars are indicated in each panel. Photographs C1–C5 are courtesy of Daigui Zhang, Jishou University.

The Xiangtoushan lineage is readily distinguished morphologically from *Fortunella*, *Papeda*, *Citrus*, and other allied genera within the citrus complex. It possesses a stable and unique combination of vegetative, floral, fruit, and seed characters (**Figure 4**). In contrast to *Citrus* and *Fortunella*, which typically have straight petioles and fully expanded petals at anthesis, the Xiangtoushan lineage has distinctly articulated petioles. The spines at the articulations are usually slightly downwardly curved or slightly falcate. Its petals persist at anthesis and form a semi-closed campanulate to tubular structure. As a result, the flowers remain incompletely expanded throughout anthesis.

Floral characters further distinguish this lineage from allied genera. *Hongdaicitrus* consistently has 19–20 stamens. The filaments of the outer, middle, and inner whorls are distinctly unequal in length and arranged in a graded series. The ovary is consistently 5-locular, with a short, stout style and a conical stigma. By contrast, *Fortunella*, *Citrus*, *Papeda*, and *Poncirus* typically have subequal filaments, slender styles, and stigmas that are hemispherical or discoid (**Table 1**; **Figure 4**).

**Table 1:** Diagnostic morphological characters distinguishing *Hongdaicitrus* from *Citrus*, *Papeda* and *Fortunella*.

Character	Citrus*	Fortunella*	Papeda*	*Hongdaicitrus*
Life form	Small tree	Small tree or shrub	small tree or shrub	Small tree or shrub; branches slender and elongate
Petiole articulation	usually present and straight	usually present and straight	usually present	articulation distinct, and the jointed region is often bent
Winged petiole	absent to broadly winged	absent to narrowly winged	broadly winged	nearly wingless
Flower	solitary axillary or several flowers in clusters	solitary axillary or several flowers in clusters	solitary or several flowers in clusters	solitary axillary
Corolla	fully spreading	mostly fully spreading	fully spreading	corolla subclosed, tubular to campanulate, lobes slightly recurved
Androecium	numerous (usually >20); filaments equal	15–20; filaments equal	stamens 16–18 or more; filaments free or weakly connate	19–20; filaments unequal, graded
Ovary locule	many, usually 7–15	3–6	usually 8–13	5
Style & stigma	style slender, 3–4× as long as stigma; stigma hemispheric to discoid	style as long as stigma to 2×; stigma hemispheric	style slender and exserted; stigma large, hemispherical to discoid, sometimes shallowly lobed	style short, stout, distinctly shorter than stigma; stigma conical
Fruit shape	globose or depressed globose	globose or ellipsoid	globose, depressed-globose	globose
Juice vesicles	often with slender stalks	spindle shaped or nearly globose	usually short or poorly developed	absent or strongly reduced
Seeds	relatively small; testa milky white, smooth to ribbed	small, ovate, acute; plump, smooth; testa milky white	seeds large, cuneate to ovate or ellipsoid; testa thick, milky white to brownish, smooth or finely striate	larger, ovoid to cuneate ellipsoid; testa brown, smooth or finely striate
Flowering period	April to May	March to May	March to May	January and September (observed in 2025)
Fruiting period	October to December	October to December	mostly from October to December or from October to January of the following year	January and November (observed in 2025)

*Comparative data from (Zhang et al., 2008); (Wang et al., 2022a).

Fruit and seed characters constitute particularly important diagnostic features of the Xiangtoushan lineage. Its fruits have a markedly thickened pericarp, and the juice vesicles are absent or almost completely reduced. At maturity, each locule is densely filled with numerous relatively large seeds, which are closely arranged around the central axis and form a compact seed cluster (**Figure 4**). Notably, the seed morphology of the Xiangtoushan lineage shows some similarity to that of certain *Papeda* taxa, such as *P. ichangensis*. Both have relatively large and irregularly shaped seeds. However, they differ clearly in seed coat color, leaf structure, and fruit internal structure. The Xiangtoushan lineage has a brown seed coat, whereas *P. ichangensis* has a milky-white seed coat. *Papeda* taxa usually have conspicuous winged petioles, with the wing often nearly as long as or longer than the leaf blade, whereas the Xiangtoushan lineage has nearly wingless petioles. In fruit internal structure, *Papeda* taxa retain a small amount of pulp tissue or reduced juice vesicles, whereas the juice vesicles of the Xiangtoushan lineage are absent or almost completely reduced. These differences indicate that, although the Xiangtoushan lineage resembles some *Papeda* taxa in seed morphology, its overall combination of morphological characters is clearly distinct from *Papeda* and other allied citrus genera.

In addition, field observations in 2025 recorded two reproductive episodes in the Xiangtoushan lineage, with flowering and fruiting observed in January and a second flowering episode in September followed by fruiting in November. Whether this pattern recurs consistently among years requires longer-term monitoring. Together with the distinctive morphological characters described above, these preliminary phenological observations facilitate diagnosis of the Xiangtoushan lineage relative to the sampled *Citrus*, *Fortunella*, and *Papeda* taxa. When evaluated alongside the phylogenomic and genome-wide evidence, this diagnosability supports the recognition of *Hongdaicitrus* under a segregate, phylogenetically informed generic framework.

**Figure 5:**
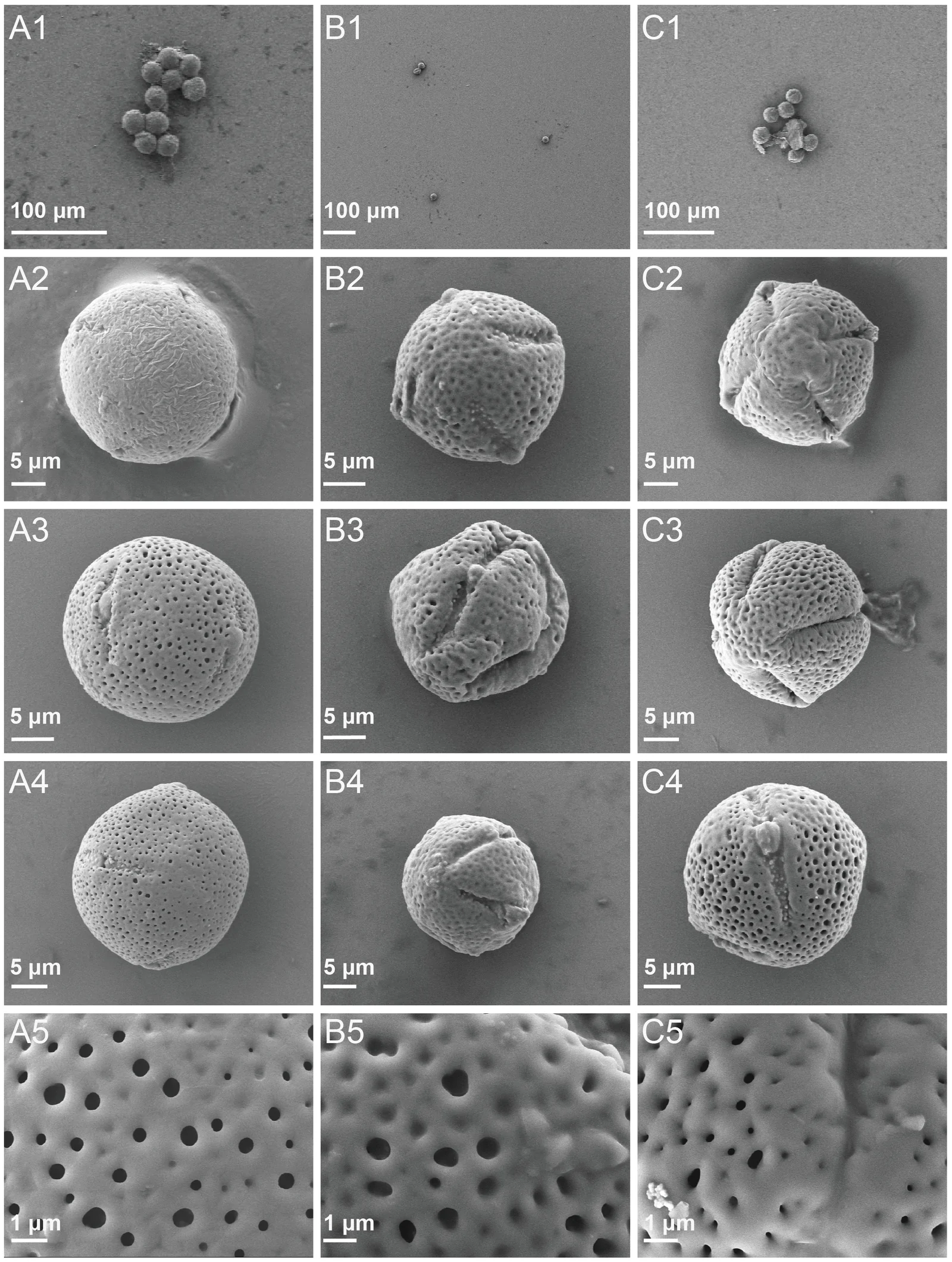
Scanning electron microscopic observation of the pollen morphology. **(A)** *Hongdaicitrus xiangtoushanensis*; **(B)** *Fortunella japonica*; **(C)** cultivated Citrus. Rows 1–5 show ensemble view, polar view, equatorial view, germination sulcus, and pollen exine ornamentation, respectively. Ensemble views show pollen grains dispersed on the stub; polar views show the arrangement of apertures from the apical region; equatorial views show the overall pollen shape; germination sulcus views show the opening morphology, depth, and width of the sulcus; high-magnification views show pollen exine ornamentation.

Pollen micromorphology also revealed diagnostic differences among the examined taxa. Scanning electron microscopy showed that pollen grains of *H. xiangtoushanensis*, *F. japonica*, and cultivated *Citrus* were broadly similar in overall shape, being nearly spheroidal to spheroidal, but differed clearly in the development of germination sulci and exine ornamentation (**Figure 5**). In *H. xiangtoushanensis*, pollen grains were nearly spheroidal to spheroidal in both polar and equatorial views and had four germination sulci. However, these sulci were less strongly developed than those of *F. japonica* and cultivated *Citrus*, with shallower and less distinct outlines. The exine surface of *H. xiangtoushanensis* was densely perforate to finely foveolate, with small and relatively uniform lumina. By contrast, *F. japonica* and cultivated *Citrus* showed more conspicuous germination sulci and coarser foveolate-reticulate exine ornamentation, with larger and more irregular lumina. This observation is consistent with previous reports that representative *Fortunella* species generally have spherical pollen grains with 4–5 germination sulci and reticulate exine ornamentation characterized by shallow foveolate lumina and irregular perforations (Wang et al., 2022b). These differences, particularly in germination sulcus development and exine ornamentation, provide additional micromorphological evidence for distinguishing *Hongdaicitrus* from closely related citrus allies.

### 3.4 Plastome features and gene content

**Figure 6:**
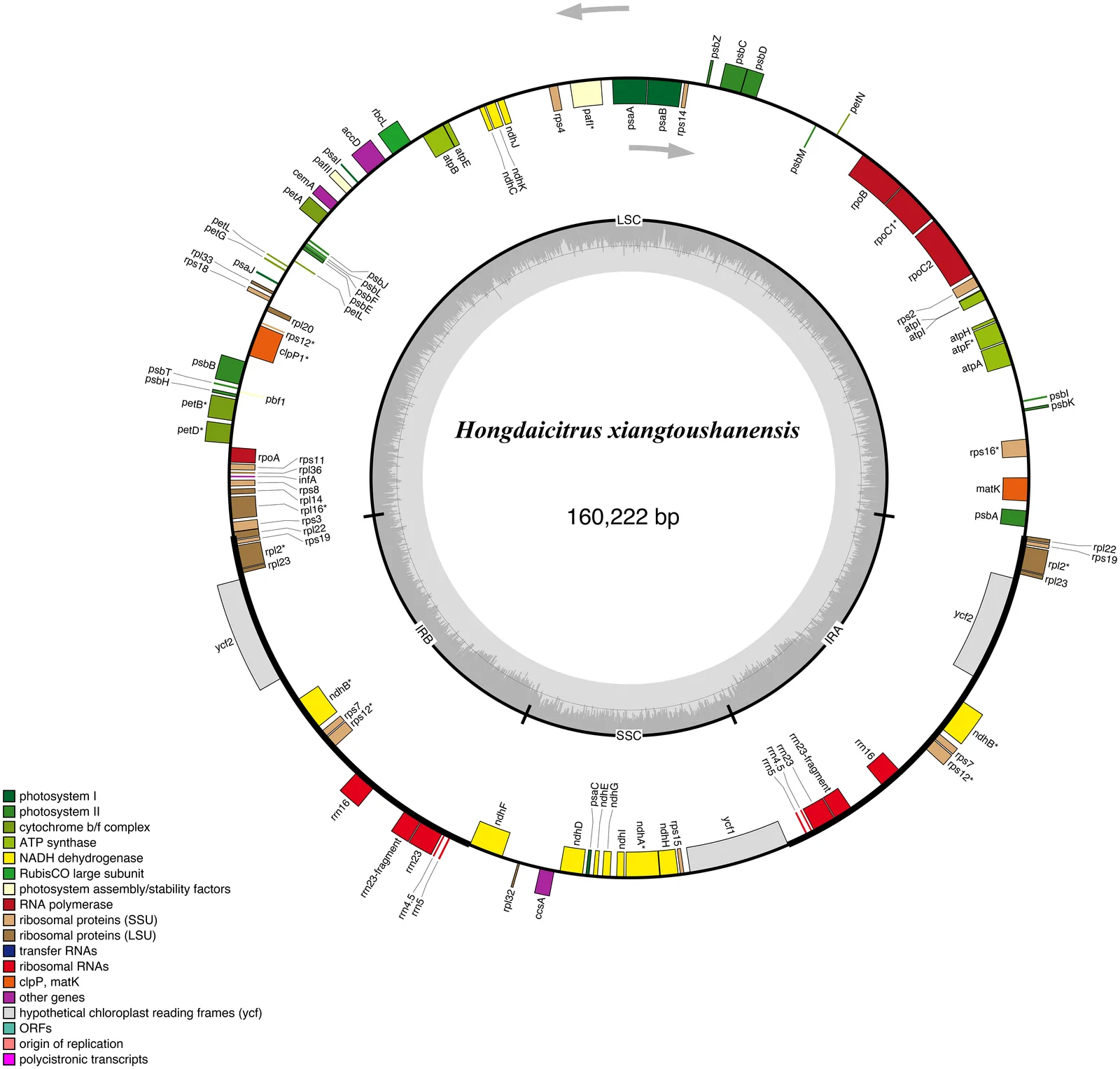
Chloroplast genome map of *Hongdaicitrus xiangtoushanensis*. Arrows indicate the transcriptional directions of genes located inside and outside the circle. Genes are color-coded according to functional group. In the inner circle, dark gray indicates GC content, and light gray indicates AT content. IR, inverted repeat; LSC, large single-copy region; SSC, small single-copy region.

The complete chloroplast genome of *Hongdaicitrus xiangtoushanensis* is 160, 222 bp in length (**Figure 6**) and exhibits a typical quadripartite structure, consisting of a large single-copy (LSC) region, a small single-copy (SSC) region, and a pair of inverted repeat regions (IRa and IRb). Comparisons among three individuals (XTS01–XTS03) reveal a high degree of consistency in plastome size, structure, and GC content, indicating strong intraspecific stability of chloroplast genome features. Detailed plastome characteristics, including genome size, lengths of the structural regions, gene content, and GC composition, are summarized in **Table 2**. Representative species of *Fortunella* and *Citrus* are also included in **Table 2** to provide a comparative framework for evaluating plastome features of *Hongdaicitrus*.

**Table 2:** Comparison of plastome size, structure, and gene content among *Fortunella*, *Citrus*, and the newly recognized genus *Hongdaicitrus*.

Species	*F. venosa*	*F. japonica*	*F. hindsii*	*C. reticulata* cv. Succosa	*C. tachibana*	XTS01	XTS02	XTS03
Genome size (bp)	160, 265	160, 229	160, 248	160, 699	160, 600	160222	160222	160222
Large single-copy (LSC, bp)	87, 597	87, 564	87, 574	87, 918	87, 771	87342	87542	87543
Small single-copy (SSC, bp)	18, 732	18, 721	18, 728	18, 801	18, 835	18730	18730	18731
Inverted repeat (IR, bp)	26, 968	26, 972	26, 973	26, 990	26, 997	26975	26975	26974
Gene	134	134	134	134	132	132	133	133
Protein-coding genes	89	89	90	87	87	89	92	92
rRNA	8	8	8	8	8	6	8	8
tRNA	37	37	37	37	37	37	37	37
GC content (%) Total genome	38.4	38.4	38.4	38.4	38.4	38.4	38.4	38.4
GC content (%) LSC	36.8	36.7	36.7	36.8	36.8	36.8	36.8	36.8
GC content (%) SSC	33.2	33.2	33.2	33.1	33.1	33.2	33.4	33.2
GC content (%) IR	43	42	42.9	42.9	42.9	42.9	42.9	42.9
GenBank accession	MZ457935	MN495932	OM773621	MW147176	NC064140			

Three individuals of *Hongdaicitrus xiangtoushanensis* (XTS01–XTS03) are included to illustrate intraspecific consistency.

## 4 Taxonomic treatment

*Hongdaicitrus* W.B. Liao, Y.R. Wang, N. Kang, Q. Fan & W.Y. Zhao, gen. nov.

Chinese name: 宏达橘属.

Type: *Hongdaicitrus xiangtoushanensis* W.B. Liao, Y.R. Wang, N. Kang, Q. Fan & W.Y. Zhao, sp. nov.

Chinese name: 象头山宏达橘.

### 4.1 Diagnosis

*Hongdaicitrus* is closely related to but readily distinguished from *Fortunella* Swingle and *Citrus* L. by the following combination of diagnostic features: petioles articulated, often slightly curved or falcate; flowers with persistent, imbricate, connivent petals forming a semi-closed campanulate structure at anthesis; stamens usually 19–20, distinctly unequal in length and arranged in three whorls (5 longer, 9–10 of medium length, and 5 shorter); fruits with a slightly thickened pericarp and little or no flesh, with juice vesicles absent or highly reduced; and locules densely packed with up to 18 large seeds arranged radially around the central axis (**Table 1**).

Monotypic genus, endemic to South China.

### 4.2 Etymology

This genus is named in honour of Hong Da Zhang (1914–2016) to recognize his outstanding research contributions to Plant Taxonomy, Floristic Phytogeography, Ecology and other related fields. Throughout his lifetime, he published more than 400 new plant species, and successively put forward “The Origin and Development of the Cathaysian Flora (1980), published “Outline of Spermatophyta Classification” (1986), and “An Outline on the Regionalisation of the Global Flora” (1991), among other classic monographs and treatises (Liao et al., 2024). *Hongdaicitrus* is a genus endemic to South China, a highly evolutionarily divergent typical element of the South China flora that is closely allied to *Citrus* s.l., and also a typical genus of the Cathaysian flora.

### 4.3 Species

**Figure 7:**
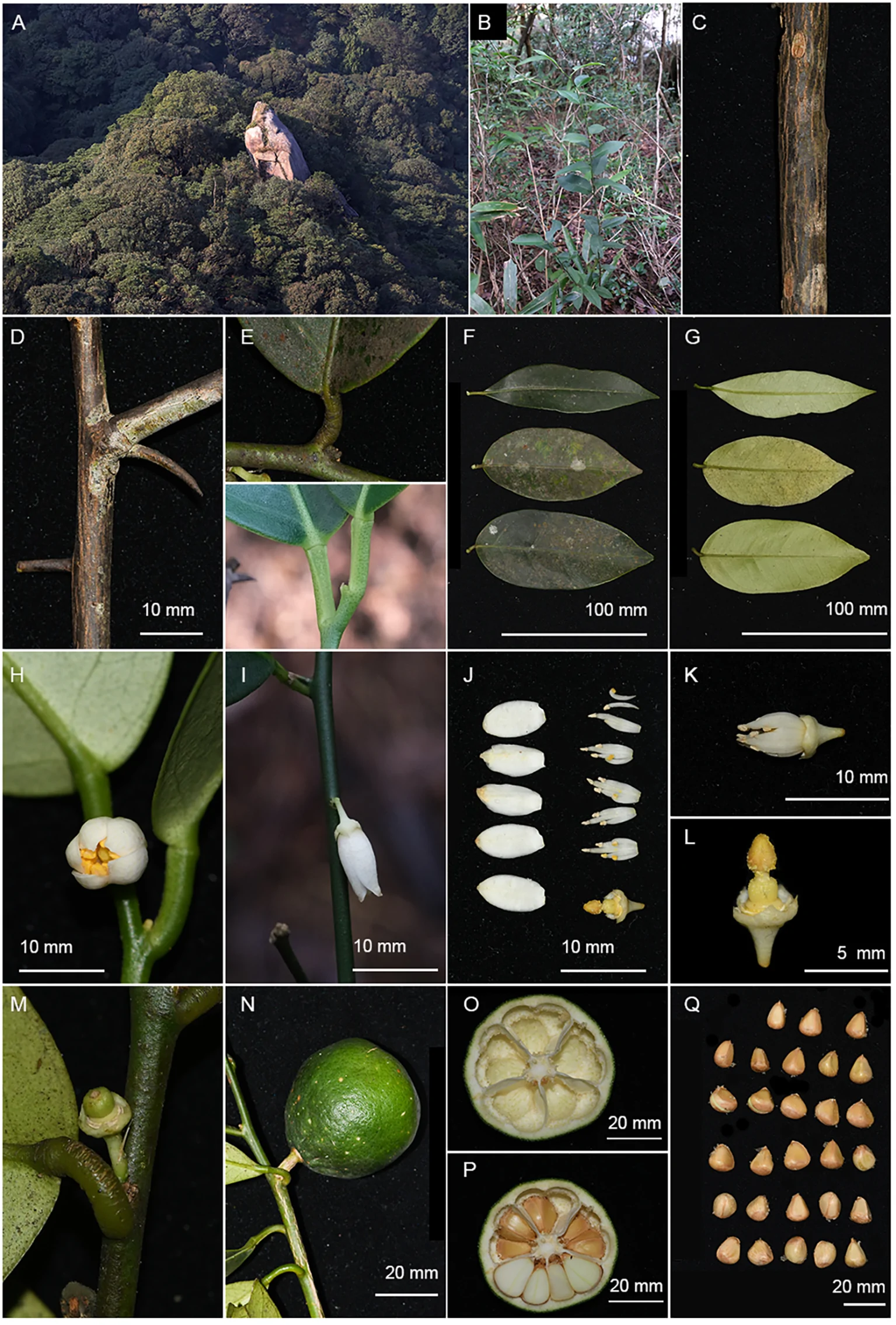
Habitat and morphological characteristics of *Hongdaicitrus xiangtoushanensis*. **(A)** General view of the habitat, growing on exposed granitic outcrops and adjacent understory of evergreen broad-leaved forest in Xiangtoushan; **(B)** Habitat view of a wild individual; **(C)** Stem; **(D)** Thorn-bearing branch; **(E)** Petiole (upper: mature leaf; lower: young leaf); **(F)** Adaxial surface of the leaf blade; **(G)** Abaxial surface of the leaf blade; **(H)** Axillary solitary flower at the early flowering stage; **(I)** Flower at anthesis; **(J)** Dissected floral organs; **(K)** Flower with perianth removed; **(L)** Close-up of the pistil, showing the ovary and style; **(M)** Young fruit attached to the branch; **(N)** Mature fruit; **(O, P)** Transverse sections of the fruit, showing the multilocular structure and arrangement of seeds; **(Q)** Seeds.

*Hongdaicitrus xiangtoushanensis* W.B. Liao, Y.R. Wang, N. Kang, Q. Fan & W.Y. Zhao, sp. nov. (**Figure 7**).

Type: CHINA. Guangdong Province, Huizhou City, Boluo County, Xiangtoushan, open or sparse woodland, in rock crevices with very shallow soil, ca. 882 m a.s.l., 23° N, 114° E, 25 Sep 2025, *Y.R. Wang, Q. Fan & Y.L. Li XTS07* (Holotype: SYS00237122).

### 4.4 Description

Small trees or shrubs, usually 0.5–3 m tall; stems slender; bark dark brown to brownish, mostly smooth, with faint longitudinal striations in some parts; each node bears a single stiff spine on one side; the spine is basally swollen, distinctly downward-curved, and falcate (**Figure 7**).

Leaves 1-foliolate, coriaceous, dark green; leaf blades elliptic to lanceolate, 8–11 cm long, 2–4 cm wide; midrib and lateral veins conspicuous, reticulate venation distinct. Apex usually acuminate to shortly acuminate, occasionally shallowly emarginate with the midrib forked near the tip; base cuneate, broadly cuneate, or subrounded. Petiole 0.8–1.0 cm long, those of young leaves green, slender, densely gland-dotted, with the articulation distinct; those of mature leaves thickened, brownish, often curved, with a wrinkled surface and relatively obscure articulation; petiole wings very narrow to almost absent.

Flowers solitary, axillary; pedicel 3–5 mm long. Petals creamy white, densely gland-dotted on the surface. Calyx cup-shaped, 5-lobed with shallow sinuses. Petals 5, 0.7–0.85 cm long, 0.35–0.45 cm wide, ovate to elliptic, thick in texture, imbricate; not fully expanded at anthesis, collectively forming a campanulate or subtubular corolla. Stamens 19–20, arranged in 2–3 whorls; 4–5 filaments connate at the base into a fascicle, forming 5 fascicles in total. Filaments unequal in length, the longest not exserted beyond the petals; the outer whorl with 5 long stamens, the middle whorl with 9–10 medium-length stamens, and the inner whorl with 5 short stamens. Ovary superior, 5-locular, short and stout cylindric; disc annular. Style short and robust, ca. 1 mm long; stigma thick, enlarged and conical.

Fruits subglobose, 3–5 cm in diam.; immature fruits dark green, becoming yellow–orange at maturity; pericarp 3–4 mm thick, surface densely gland-dotted, oil vesicles slightly raised; almost devoid of flesh, with juice vesicles highly reduced or absent (**Figure 7**).

Carpels syncarpous, 5-locular; ovary superior, with axile placentation; each locule densely packed with seeds at maturity. Seeds ovoid-conical, tapering gradually toward the hilar end and obtuse at the distal end, arranged radially within each locule and closely appressed to the central axis. Seed coat yellowish brown, smooth, with faint longitudinal striations; endosperm absent; embryo bearing two thick, fleshy, creamy white cotyledons.

### 4.5 Phenology

Phenological observations were conducted in 2025. Flowers and fruits were recorded on five individuals in January. A further flowering event was observed on ten individuals in September, followed by the observation of fruits in November. These records indicate two periods of reproductive activity during the survey year but do not yet establish a consistent annual bimodal cycle. Multi-year monitoring is required to determine the timing, duration, and interannual stability of flowering and fruiting in *Hongdaicitrus xiangtoushanensis*.

### 4.6 Additional specimens examined

Paratypes of *Hongdaicitrus xiangtoushanensis*. CHINA. Guangdong Province, Huizhou City, Boluo County, Xiangtoushan, open or sparse woodland, growing in rock crevices with very shallow soil, ca. 882 m a.s.l., 23.27° N, 114.38° E: 19 January 2025, Y.R. Wang, Q. Fan & Y.L. Li, XTS01 (SYS00237125), XTS02 (SYS00237129), XTS03 (SYS)!; 11 September 2025, Y.R. Wang, Q. Fan & Y.L. Li, XTS104301 (SYS00237124), XTS104302 (SYS00237137), XTS104303 (SYS00237136), XTS104304 (SYS)!, XTS104305 (SYS00237134), XTS104306 (SYS00237133), XTS104307 (SYS00237135), XTS104308 (SYS)!, XTS104309 (SYS00237132), XTS1043010 (SYS)!, XTS1043011 (SYS)!, XTS1043012 (SYS00237131), XTS1043013 (SYS00237130); 13 December 2025, Y.R. Wang, Q. Fan & Y.L. Li, XTS04 (SYS00237126), XTS05 (SYS00237127), XTS06 (SYS00237128), XTS08 (SYS00237123).

Comparative specimens examined. The following herbarium specimens were examined for morphological comparison with Hongdaicitrus: *Citrus reticulata* (mangshanyeju), HIB HIB0208276; *Fortunella japonica*, NAS NAS00404635; NAS00583222; NAS00586608; NAS00404633; NAS00404628; IBK IBK00108685; IBK00108683; IBK00108682; IBK00108681; IBK00108684; IBK00108689; IBK00108690; IBK00108688; *Fortunella hindsii*, PE01808148; 01377852; 00690745; 00690746; 00690747; 00690743; 00693170; *Papeda ichangensis*, PE00626702; 0515960; 02028676; 02028677; KUN0515958; 0515962; 1331809; 0515967; IBK00434897; 00408109.

### 4.7 Distribution and habitat

*Hongdaicitrus xiangtoushanensis* is currently known only from Xiangtoushan National Nature Reserve, Guangdong Province, South China. It occurs on exposed granitic outcrops and in open or sparsely wooded areas on hillsides and hilltops at approximately 600–900 m a.s.l. Individuals are typically rooted in narrow rock fissures containing extremely shallow, apparently nutrient-poor soil. These microsites provide limited rooting space and are likely subject to rapid drainage, periodic water limitation, high solar exposure, and pronounced fluctuations in temperature and moisture (Poot et al., 2012; Ottaviani et al., 2016). Its coriaceous leaves may contribute to water conservation under exposed conditions, while establishment in rock fissures may enable roots to exploit localized soil and moisture retained within fractured bedrock. These possible functional adaptations remain to be tested through analyses of soil properties, root architecture, leaf anatomy, and plant water-use physiology.

### 4.8 Conservation status

*Hongdaicitrus xiangtoushanensis* is provisionally assessed as Critically Endangered (CR) following the IUCN Red List Categories and Criteria (IUCN, 2012). It is currently known from a single locality in Xiangtoushan National Nature Reserve and is restricted to granitic outcrops and narrow rock fissures containing extremely shallow soil. The limited soil volume and water-storage capacity of these microsites may increase water stress during prolonged drought, particularly affecting seedling survival and natural recruitment. Conversely, extreme rainfall may erode the shallow substrate, destabilize established plants, and reduce the availability of suitable establishment sites. Changes in canopy cover may alter local light, temperature, and moisture conditions, while physical disturbance of occupied outcrops could directly damage individuals or destroy their specialized microhabitats. Fruit and seed damage may reduce reproductive output, and stem-boring damage caused by unidentified insects was observed in several individuals (**Supplementary Figure 2**), potentially weakening stems and reducing plant vigour. Because all known individuals are concentrated within a single, spatially restricted habitat, a localized extreme event could affect a substantial proportion of the known population. Although long-term demographic data are not yet available to demonstrate an ongoing decline, the restricted distribution and habitat specialization indicate a high risk of rapid population loss.

The known population occurs within the core zone of the reserve and therefore receives legal protection. Nevertheless, a species-specific management programme is recommended (Ren et al., 2012; Yang et al., 2020). Priority actions should include mapping and long-term monitoring of all known individuals, protection of occupied granitic microhabitats, assessment of reproductive success and natural recruitment, identification and monitoring of stem-boring insects, and investigation of pollination, seed viability, germination, and seedling establishment. Ex situ conservation through regulated seed collection, living collections, tissue culture, and germplasm storage should also be established. Population-genomic data should be obtained before any reinforcement, translocation, or reintroduction is undertaken. The conservation assessment should be updated as further information becomes available on population size, distribution, demographic trends, and threat severity.

## 5 Discussion

### 5.1 Phylogenetic independence of the Xiangtoushan lineage and generic delimitation under *Citrus* s.l.

Phylogenetic analyses consistently indicate that the Xiangtoushan lineage represents an evolutionarily independent clade within the sampled framework of *Citrus* sensu lato. Although the lineage was recovered as sister to *Fortunella*, it was not nested within *Fortunella* or any of the other traditional genus-level lineages sampled here. The Xiangtoushan lineage formed a strongly supported monophyletic clade in both the ML/BI analyses of 83 plastid CDS and the ML analysis of 5, 873 single-copy nuclear orthologues. It was stably resolved as sister to *Fortunella* and clearly separated from *Citrus* s.s., *Papeda* and *Poncirus* (**Figures 1A, B**; **Supplementary Figure 1**). The ASTRAL analysis of 5, 865 individual nuclear gene trees recovered the same sister-group relationship, with the focal topology receiving the highest local quartet support (**Figure 2**; **Supplementary Table 5**). The high site concordance factor supported this relationship, whereas the moderate gene concordance factor indicated substantial heterogeneity among individual gene trees. Thus, despite discordance among some loci, the *Hongdaicitrus–Fortunella* relationship represents the predominant nuclear phylogenetic signal. This agreement between the plastid and nuclear datasets with respect to the focal sister-group relationship supports the stability of its phylogenetic placement under the present sampling framework. Genome-wide resequencing analyses further support this interpretation. The three Xiangtoushan individuals formed a compact genetic cluster in PCA and pairwise 1 − IBS analyses, clearly separated from sampled *Fortunella*, *Citrus*, *Papeda*, and *Poncirus* accessions (**Figures 3A–C**). In particular, the genetic distances between XTS and *Fortunella* were markedly greater than within-XTS distances, indicating that the Xiangtoushan lineage does not represent an intraspecific variant of the sampled wild *Fortunella hindsii* accessions. Thus, the phylogenomic and genome-wide results jointly support the evolutionary independence of the Xiangtoushan lineage (Wang et al., 2022b; Zhu et al., 2022).

The gene-tree heterogeneity detected here is consistent with the complex evolutionary history of the citrus complex, in which incomplete lineage sorting and historical gene flow can generate discordant relationships among loci (Maddison, 1997; Degnan and Rosenberg, 2009; Wu et al., 2018). However, gene-tree discordance alone does not demonstrate a hybrid origin. D-statistics detected greater allele sharing between *Fortunella* and *Papeda* than between *Hongdaicitrus* and *Papeda*, but did not detect preferential allele sharing between the latter two lineages (**Figure 3D**; **Supplementary Table 6**). Thus, the present data do not support a hybrid origin of *Hongdaicitrus* involving the sampled *Papeda* lineage, although introgression involving unsampled or extinct lineages cannot be excluded (Tricou et al., 2022).

Taxon sampling was designed to evaluate the phylogenetic position of the Xiangtoushan lineage within *Citrus* s.l., with particular emphasis on *Fortunella*, which was recovered as its closest relative in both the plastid and nuclear analyses. The plastid dataset included multiple accessions of *F. hindsii*, *F. venosa*, and *F. japonica*, representing both wild and cultivated kumquat taxa, together with representatives of the Australasian citrus-related genera *Clymenia*, *Eremocitrus*, and *Microcitrus* (**Supplementary Table 1**; **Figure 1A**). The nuclear dataset likewise included representatives of *Fortunella*, *Microcitrus*, and *Eremocitrus* (**Supplementary Table 1**; **Figure 1B**). Collectively, these datasets encompass the principal traditional lineages of *Citrus* s.l. directly relevant to the present generic comparison and provide a focused, although not exhaustive, framework for evaluating the placement of the Xiangtoushan lineage.

This result has important implications for citrus systematics. Multiple phylogenetic studies indicate that *Citrus* s.l. encompasses several long-recognized and traditionally segregated lineages, including *Fortunella*, *Poncirus*, *Papeda*, and *Citrus* s.s (Wang et al., 2018; Zhu et al., 2019, 2022). The consistent recovery of the Xiangtoushan lineage as sister to, rather than nested within, *Fortunella* demonstrates its phylogenetic independence but does not, by itself, determine taxonomic rank. Its recognition as *Hongdaicitrus* is instead based on the combined evidence of consistent phylogenetic placement across the plastid and nuclear datasets, quantitative genome-wide differentiation, and a distinctive suite of vegetative, floral, fruit, and seed characters discussed below. Placement of this lineage within the monophyletic true citrus group establishes its common ancestry with other citrus lineages, but does not require that all constituent lineages be assigned to a single genus (Bayer et al., 2009; Wu et al., 2018). Under the segregate framework adopted here, recognition of *Hongdaicitrus* is not an isolated addition of a new genus; it entails evaluating *Fortunella*, *Papeda*, *Poncirus*, and other comparably differentiated lineages according to the same integrative criteria and, where supported, recognizing or recircumscribing them accordingly (Humphreys and Linder, 2009). It also has implications for the delimitation of *Citrus* s.s. Accordingly, the present treatment provides a phylogenetically informed genus-level placement of the Xiangtoushan lineage, whereas a comprehensive segregate classification of the entire true citrus group will require broader systematic and nomenclatural reassessment.

Further investigation should combine complementary molecular markers with advanced genomic approaches. Plastid haplotypes could clarify cytoplasmic lineage histories, whereas low-copy nuclear loci and genome-wide SNPs could resolve biparentally inherited evolutionary relationships. Nuclear microsatellites or targeted SNP panels could support population-level assessments of genetic diversity, relatedness, and inbreeding (Zhu et al., 2022; Wang et al., 2023). Future taxonomic sampling should prioritize geographically representative, voucher-documented wild populations because many cultivated citrus accessions have admixed ancestry that has subsequently been maintained through apomixis or clonal propagation (Mabberley, 2004; Wu et al., 2014, 2018; Wang et al., 2017). Chromosome-level, haplotype-resolved genome assemblies and expanded resequencing of *Hongdaicitrus* and its close relatives would enable comparisons of structural variation and demographic history. With broader population and taxonomic sampling, local phylogenetic inference, topology weighting, admixture graphs, and phylogenetic-network analyses could further distinguish incomplete lineage sorting from complex historical introgression, while graph-based pangenomes could identify lineage-specific genomic changes associated with diversification (Wu et al., 2018, 2021; Wang et al., 2022a; Huang et al., 2023).

### 5.2 Morphological diagnosability and the persistence of discrete lineages in a reticulate complex

The widespread occurrence of hybridization, backcrossing, and apomixis in the citrus complex has complicated generic delimitation (Wang et al., 2017, 2022a), but it has not eliminated the taxonomic value of stable morphological and phenological traits in traditional classifications. For instance, *Fortunella* has historically been distinguished by several characters, including few ovary locules (3–9), usually two ovules per locule, a small tree habit, and prolonged summer flowering (Tanaka, 1954a; Swingle and Reece, 1967; Zhou and Ye, 2009). Similarly, *Poncirus* is diagnosed by its deciduous habit, palmately trifoliolate leaves, numerous stamens with free filaments, and densely pubescent ovaries and mature fruits (Zhang et al., 2008; Peng et al., 2020). These examples show that stable diagnostic traits remain useful for recognizing discrete lineages, even in a group with extensive reticulate evolution.

In this context, *Hongdaicitrus* exhibits a consistent combination of vegetative, floral, fruit, and seed characters that distinguishes it from *Citrus*, *Fortunella*, *Papeda*, and other allied lineages (Deng et al., 2008). Its principal diagnostic characters include well-developed, downward-curved spines; pendulous flowers borne on reflexed pedicels; a semi-closed, campanulate to tubular corolla that persists at anthesis; 19–20 stamens with markedly unequal filament lengths and filaments basally connate into five bundles; and a short, stout style with an obturbinately conical stigma. The fruit has a markedly thickened pericarp, almost no pulp, and absent or highly reduced juice vesicles, while each locule contains numerous relatively large, triangular-conical seeds densely arranged around the central axis. Rather than any single character, this stable combination provides the morphological diagnosis of the lineage.

Fruit and seed characters provide particularly strong diagnostic evidence. The fruits of *Hongdaicitrus* have a thickened pericarp and lack normally developed juice vesicles, resulting in an almost fleshless internal structure. At maturity, each locule is densely packed with numerous relatively large seeds, which are arranged around the central axis and form a compact seed mass. Although these large and irregularly shaped seeds resemble those of some *Papeda* taxa, such as *P. ichangensis*, *Hongdaicitrus* differs from the sampled *Papeda* taxa in its nearly wingless petioles, brown seed coat, floral structure, and more extensive reduction of the juice vesicles. This fruit structure stands in even sharper contrast to the typical fruit architecture of *Citrus* and *Fortunella* (Wang et al., 2022a; Huang et al., 2023; Zhou and Ye, 2009). The D-statistic analyses did not detect preferential allele sharing between *Hongdaicitrus* and the sampled *Papeda* lineage (**Figure 3D**; **Supplementary Table 6**). Consequently, their partial similarity in fruit and seed morphology does not, on the current evidence, indicate a recent hybrid origin of *Hongdaicitrus*, although more complex historical introgression involving unsampled lineages cannot be excluded.

Phenological observations in 2025 recorded two periods of reproductive activity in *Hongdaicitrus*, with flowers and fruits observed in January and a further flowering event in September followed by fruiting in November. This preliminary pattern may differ from the predominantly spring-flowering pattern reported for many related *Citrus* and *Fortunella* taxa (Zhu et al., 2022; Mishra et al., 2024), but multi-year observations are required to determine its consistency and taxonomic significance. The semi-closed corolla is morphologically distinctive, although its functional significance remains unclear because the pollination biology of *H. xiangtoushanensis* was not investigated in the present study. Studies of cultivated *Citrus* indicate that animal visitation can enhance fruit set, although dependence on pollinators varies among taxa with different mating and reproductive systems (Liang et al., 2020; Monasterolo et al., 2024). Future studies combining observations of floral visitors, pollinator-exclusion experiments, and controlled self- and cross-pollinations are needed to determine its mating system, floral accessibility to pollinators, and whether pollination limitation reduces reproductive success or contributes to low natural recruitment.

Collectively, these morphological, reproductive, and phenological characters demonstrate the diagnosability of *Hongdaicitrus*. Morphology alone does not determine generic rank, but its observed diagnostic character combination, evaluated together with phylogenomic placement and genome-wide differentiation, supports recognition of *Hongdaicitrus* as a distinct genus within the reticulate citrus complex.

### 5.3 Implications of Hongdaicitrus’s discovery for Citrus systematics and conservation of wild citrus in South China

South China, part of the Cathaysian Block, underwent multiple episodes of Cretaceous tectonic extension, rifting, and magmatism that shaped a highly dissected and heterogeneous landscape (Li et al., 2014; Bonnetti et al., 2018; Shu et al., 2021). The resulting topographic and environmental heterogeneity may have promoted geographic isolation and provided diverse refugial habitats for the persistence and differentiation of plant lineages.

Recent phylogenomic and biogeographic studies indicate that several major lineages of *Citrus* s.l. occur or overlap in South-Central China, particularly around the Nanling Mountains (Heads, 2025; Huang et al., 2023). This regional concentration of citrus diversity, together with the discovery of the independently differentiated Xiangtoushan lineage in Guangdong, highlights South China as an important area for understanding the diversification of wild citrus relatives.

The discovery of *Hongdaicitrus* in Xiangtoushan National Nature Reserve, Huizhou, Guangdong Province, further emphasizes the importance of the heterogeneous montane landscapes of South China. Xiangtoushan lies in east-central Guangdong and is geographically distinct from the Nanling Mountains; these regions should therefore be regarded as separate components of the broader South China landscape rather than as a single mountain system. In south central China, the distributions of several citrus lineages converge, a zone that largely corresponds to the Nanling range (Heads, 2025). As a major watershed between the Pearl and Yangtze River systems, the topographic complexity of the Nanling may have facilitated lineage persistence and differentiation through habitat fragmentation and microhabitat heterogeneity.

The Nanling is also recognized as an important center of biodiversity and endemism. For example, a recent nationwide assessment identified the highest amphibian species richness among 567 surveyed grid cells in the Nanling Mountains (Xu et al., 2024). Although patterns documented in other organismal groups cannot be directly extrapolated to citrus, they illustrate the exceptional biological heterogeneity of this region. Together with the discovery of *Hongdaicitrus*, these observations suggest that South China may contain additional wild citrus lineages that remain insufficiently documented or taxonomically resolved. Future surveys should therefore prioritize geographically isolated, voucher-documented wild populations rather than relying predominantly on cultivated or potentially admixed accessions.

The highly restricted distribution of *H. xiangtoushanensis* also gives this discovery an immediate conservation significance. Protection of its granitic microhabitats, long-term demographic monitoring, and conservation of representative germplasm are necessary not only to preserve the species itself but also to retain a phylogenetically distinctive component of South China’s wild citrus diversity.

### 5.4 Our perspective on generic delimitation: wild lineages as anchors for a natural classification

In delimiting genera within the citrus complex, we propose that voucher-documented wild populations should serve as important reference points for developing a phylogenetically informed classification. As additional wild germplasm is identified and phylogenetically corroborated, lineages that are consistently resolved as evolutionarily distinct and possess stable combinations of diagnostic characters should be evaluated as candidates for recognition at generic rank. *Fortunella* and *Poncirus*, for instance, have long been distinguished by stable morphological and biological traits, and molecular evidence further supports their independent evolutionary histories (Bayer et al., 2009; Peng et al., 2020; Wang et al., 2023). Similarly, the Xiangtoushan lineage (*Hongdaicitrus*) constitutes a well-supported monophyletic group in plastid, concatenated nuclear, and multispecies-coalescent analyses and exhibits a stable combination of diagnostic vegetative and reproductive characters. Together with its genome-wide differentiation from the sampled *Fortunella* accessions, this integrated evidence supports its recognition at generic rank under the segregate taxonomic framework adopted here.

The adoption of this framework is not motivated by a preference for small genera or by phylogenetic topology alone. The widespread operational use of broadly circumscribed *Citrus* has a historical and practical basis, particularly in view of the complex hybrid ancestry and reproductive biology of cultivated citrus, incomplete representation of wild populations, and the value of nomenclatural continuity in horticulture and germplasm management (Ollitrault et al., 2020). Nevertheless, continuing discoveries of voucher-documented wild lineages with concordant morphological, ecological, phylogenetic, and genomic differentiation reveal evolutionary structure that may be insufficiently expressed when all such lineages are assigned to a single broadly circumscribed genus. The purpose of a segregate framework is therefore not to maximize the number of genera, but to provide explicit taxonomic recognition for independently differentiated and diagnosable evolutionary lineages while maintaining an internally coherent classification (Humphreys and Linder, 2009; Bouman et al., 2021, 2022).

Recognition of *Hongdaicitrus* consequently cannot be considered independently of the classification of the remainder of the true citrus group. Under a consistently applied segregate framework, *Fortunella*, *Papeda*, *Poncirus*, and other comparably differentiated lineages must be evaluated according to the same integrative criteria and, where supported, recognized or recircumscribed accordingly. This treatment also has implications for the delimitation of *Citrus* s.s., including the lineage containing the nomenclatural type of *Citrus*. The present study does not attempt to provide all nomenclatural combinations or a complete recircumscription of *Citrus* s.l., because such a revision would require substantially broader taxonomic sampling—particularly of voucher-documented wild populations—as well as coordinated morphological, genomic, and nomenclatural assessment. Rather, our results identify one independently differentiated lineage and explicitly acknowledge the broader classificatory consequences of recognizing it at generic rank.

Comparable evidence-based segregation has substantial precedent in angiosperm taxonomy. Integrated molecular and morphological evidence led to the division of *Phyllanthus* s.l. into ten monophyletic and morphologically interpretable genera (Bouman et al., 2021, 2022). Similarly, the polyphyletic *Acacia* s.l. was reorganized into several phylogenetically distinct genera, including *Acacia* s.s., *Vachellia*, *Senegalia*, *Acaciella*, and *Mariosousa* (Kyalangalilwa et al., 2013). In tribe Wisterieae, combined nuclear, plastid, and morphological evidence likewise supported the recognition or recircumscription of multiple diagnosable genera (Compton et al., 2019). Continuing debates in groups such as Magnoliaceae further illustrate that generic delimitation must balance monophyly, diagnosability, evolutionary differentiation, nomenclatural stability, and practical utility rather than being determined by topology or genus size alone (Aldaba Núñez et al., 2024; Jiang et al., 2024; Li et al., 2024; Muñoz-Rodríguez et al., 2023). These examples do not imply that segregation is universally preferable; rather, they demonstrate that it is an established taxonomic solution when the resulting lineages are phylogenetically supported, morphologically diagnosable, and capable of forming a coherent classification.

Accordingly, recognition of *Hongdaicitrus* is not proposed as an isolated act of taxonomic splitting or as a complete resolution of generic boundaries across the citrus complex. It represents the application of an explicitly stated, phylogenetically informed segregate framework in which comparably differentiated lineages are evaluated according to the same integrative criteria. A comprehensive implementation of this framework across the true citrus group remains an important objective for future systematic and nomenclatural revision.

## Data Availability

The raw whole-genome resequencing data generated in this study have been deposited in the Genome Sequence Archive (GSA) of the National Genomics Data Center (NGDC) under BioProject accession PRJCA059069. Individual BioSample and run accession numbers are provided in **Supplementary Tables 2** and **3**. The complete chloroplast genome assemblies and annotation files of Hongdaicitrus xiangtoushanensis (XTS01, XTS02, and XTS03) are available through Figshare link: https://figshare.com/s/9abf113984ac07a29d9c.
