# Supplementary material for: Integrative morphological, palynological, and phylogenomic evidence supports the recognition of Hongdaicitrus as a new genus within Citrus sensu lato
Source: Front Plant Sci. 2026 Sep 3;17:1905317. doi: 10.3389/fpls.2026.1905317 (PMC13582474; doi:10.3389/fpls.2026.1905317)
Supplement: Supplementary file 1 [file DataSheet1.docx]

Supplementary Material

# 1 Supplementary Figures and Tables

## 1.1 Supplementary Figures

**Supplementary Figure S1. Bayesian-inference phylogeny of *Citrus* s.l. inferred from a concatenated alignment of 83 plastid protein-coding sequences (CDS).** The phylogeny was reconstructed using MrBayes, with posterior probabilities shown at the nodes. Traditional genus-level lineages used for comparison in this study are indicated by coloured background boxes. Names in parentheses indicate the corresponding combinations under a broadly circumscribed *Citrus* treatment. “Australasian citrus relatives” denotes a geographic assemblage comprising the sampled *Clymenia*, *Eremocitrus*, and *Microcitrus* taxa rather than a single genus. The three accessions of *Hongdaicitrus xiangtoushanensis* (XTS01–XTS03) form a strongly supported monophyletic clade that is resolved as sister to *Fortunella* rather than nested within it. *Atalantia buxifolia* and *A. kwangtungensis* were used as outgroups. The scale bar indicates substitutions per site.

**Supplementary Figure 2. Stem damage consistent with boring by unidentified insects in wild individuals of** ***Hongdaicitrus xiangtoushanensis*.** (A) Longitudinal splitting and bark loss on an affected stem. (B) Cross-sectional view showing an internal gallery and partial hollowing of the stem. (C) Extensive internal tunnelling and deterioration of woody tissues. The causal insect has not yet been taxonomically identified.

## 1.2 Supplementary Tables

**Supplementary Table1 GenBank accession numbers and taxa of 35 plastid genomes downloaded from NCBI and used in phylogenetic analyses.**

| GenBank accession no. | Taxon | Name under *Citrus* s.l. |
| --- | --- | --- |
| XTS3 | ***Hongdaicitrus xiangtoushanensis*** | ***H. xiangtoushanensis*** |
| XTS2 | ***Hongdaicitrus xiangtoushanensis*** | ***H. xiangtoushanensis*** |
| XTS1 | ***Hongdaicitrus xiangtoushanensis*** | ***H. xiangtoushanensis*** |
| MZ408683 | *Atalantia buxifolia* | *Atalantia buxifolia* |
| MH329190 | *Atalantia kwangtungensis* | *Atalantia kwangtungensis* |
| KJ865401 | *Citrus × aurantifolia* | *Citrus × aurantifolia* |
| MT702983 | *Citrus × aurantium* | *Citrus × aurantium* |
| ON065547 | *Citrus × junos* | *Citrus × junos* |
| NC_064135 | *Citrus × junos* | *Citrus × junos* |
| ON169956 | *Citrus × limon* | *Citrus × limon* |
| MT880608 | *Citrus × limon* | *Citrus × limon* |
| ON911462 | *Citrus × sinensis* | *Citrus × sinensis* |
| NC_008334 | *Citrus × sinensis* | *Citrus × sinensis* |
| ON872191 | *Citrus indica* | *Citrus indica* |
| NC_065753 | *Citrus indica* | *Citrus indica* |
| SRR17013819 | *Citrus mangshanensis* | *Citrus mangshanensis* |
| SRR17013818 | *Citrus mangshanensis* | *Citrus mangshanensis* |
| SRR17013817 | *Citrus mangshanensis* | *Citrus mangshanensis* |
| ON169957 | *Citrus maxima* | *Citrus maxima* |
| NC_034290 | *Citrus maxima* | *Citrus maxima* |
| MT106673 | *Citrus medica* | *Citrus medica* |
| OM773632 | *Citrus reticulata* | *Citrus reticulata* |
| OM773615 | *Citrus reticulata* | *Citrus reticulata* |
| MW147176 | *Citrus reticulata* | *Citrus reticulata* |
| ON065552 | *Citrus tachibana* | *Citrus tachibana* |
| NC_064140 | *Citrus tachibana* | *Citrus tachibana* |
| LC794896 | *Clymenia polyandra* | *citrus polyandra* |
| OR664168 | *Eremocitrus glauca* | *citrus glauca* |
| LC794891 | *Eremocitrus glauca* | *citrus glauca* |
| OM773626 | *Fortunella hindsii* | *Citrus hindsii* |
| OM773621 | *Fortunella hindsii* | *Citrus hindsii* |
| OM773617 | *Fortunella hindsii* | *Citrus hindsii* |
| OM773630 | *Fortunella japonica* | *Citrus japonica* |
| OM773623 | *Fortunella japonica* | *Citrus japonica* |
| OM773616 | *Fortunella japonica* | *Citrus japonica* |
| OM773613 | *Fortunella japonica* | *Citrus japonica* |
| OM773611 | *Fortunella japonica* | *Citrus japonica* |
| OM773610 | *Fortunella japonica* | *Citrus japonica* |
| MN495932 | *Fortunella japonica* | *Citrus japonica* |
| OM773628 | *Fortunella venosa* | *Citrus venosa* |
| OM773614 | *Fortunella venosa* | *Citrus venosa* |
| MZ457935 | *Fortunella venosa* | *Citrus venosa* |
| NC_058999 | *Microcitrus australasica* | *citrus australasica* |
| MZ929414 | *Microcitrus australasica* | *citrus australasica* |
| NC_053573 | *Papeda hongheensis* | *Citrus hongheensis* |
| MT880607 | *Papeda ichangensis* | *Citrus ichangensis* |
| MK250977 | *Poncirus polytrifolia* | *Citrus polytrifolia* |
| OM773612 | *Poncirus trifoliata* | *Citrus trifoliata* |

**Supplementary Table2 Datasets and accession numbers used for single-copy nuclear phylogenetic analyses**

| Accession ID | Primary reference | Taxon | Name under *Citrus* s.l. | Data source |
| --- | --- | --- | --- | --- |
| SRR14766314 | (Wang et al., 2018) | *Fortunella japonica* | *Citrus japonica* | NCBI |
| EM04 | This study | *Papeda ichangensis* | *Citrus ichangensis* | This study |
| KBG4 | This study | *Poncirus polyandra* | *Citrus polyandra* | This study |
| SRR25399167 | (Liang et al., 2024) | *Citrus tachibana* | *Citrus tachibana* | NCBI |
| XTS01 | This study | *Hongdaicitrus xiangtoushanensis* | *Hongdaicitrus xiangtoushanensis* | This study |
| XTS02 | This study | *Hongdaicitrus xiangtoushanensis* | *Hongdaicitrus xiangtoushanensis* | This study |
| XTS03 | This study | *Hongdaicitrus xiangtoushanensis* | *Hongdaicitrus xiangtoushanensis* | This study |
| SRR14482355 | (Wu et al., 2021) | *Citrus ryukyuensis* | *Citrus ryukyuensis* | NCBI |
| SRR17027002 | (Huang et al., 2023) | *Citrus glauca* | *Citrus glauca* | NCBI |
| SRR17035236 | (Huang et al., 2023) | *Citrus australasica* | *Citrus australasica* | NCBI |
| SRR6188449 | (Wu et al., 2018) | *Citrus australis* | *Citrus australis* | NCBI |
| SRR3948493 | (Wang *et al*., 2017） | *Citrus indica* | *Citrus indica* | NCBI |
| GCA_002013935.1 | (Wang *et al*., 2017） | *Atalantia buxifolia* | *Atalantia buxifolia* | NCBI |
| GCA_004802465.1 | (Wu et al., 2025) | *Fortunella hindsii* | *Citrus hindsii* | NCBI |
| GCA_018350135.1 | (Huang et al., 2021) | *Poncirus trifoliata* | *Citrus trifoliata* | NCBI |
| GCA_002013955.2 | (Wang *et al*., 2017） | *Citrus medica* | *Citrus medica* | NCBI |
| GCA_046463855.1 | (Liu *et al*., 2025) | *Citrus mangshanyeju* | *Citrus mangshanyeju* | NCBI |
| PRJCA019821 | (Liu *et al*., 2025) | *Citrus mangshanensis* | *Citrus mangshanensis* | GWH |
| GWHDODL00000000 | (Huang et al., 2023) | *Citrus maxima* | *Citrus maxima* | GWH |

**Supplementary Table3 Samples used for genome-wide resequencing analyses.**

| Sample | Taxonomic group | ScientificName | Data source |
| --- | --- | --- | --- |
| NL3 | *Citrus* | *Citrus reticulata* | This study |
| NL4 | *Citrus* | *Citrus reticulata* | This study |
| NL5 | *Citrus* | *Citrus reticulata* | This study |
| NL6 | *Citrus* | *Citrus reticulata* | This study |
| SRR5796635 | *Citrus* | *Citrus reticulata* |  |
| SRR5796818 | *Citrus* | *Citrus reticulata* |  |
| MS01 | *Citrus* | *Citrus reticulata* | This study |
| MS02 | *Citrus* | *Citrus reticulata* | This study |
| NL1 | *Citrus* | *Citrus reticulata* | This study |
| NL2 | *Citrus* | *Citrus reticulata* | This study |
| SRR19016679 | *Fortunella* | *F.* *hindsii* | Wang et al.2023 |
| SRR22345194 | *Fortunella* | *F. hindsii* | Wang et al.2023 |
| SRR22345195 | *Fortunella* | *F. hindsii* | Wang et al.2023 |
| SRR22345196 | *Fortunella* | *F. hindsii* | Wang et al.2023 |
| SRR22345197 | *Fortunella* | *F. hindsii* | Wang et al.2023 |
| SRR22345198 | *Fortunella* | *F. hindsii* | Wang et al.2023 |
| SRR22345199 | *Fortunella* | *F. hindsii* | Wang et al.2023 |
| SRR22345200 | *Fortunella* | *F. hindsii* | Wang et al.2023 |
| SRR22345201 | *Fortunella* | *F. hindsii* | Wang et al.2023 |
| SRR22345205 | *Fortunella* | *F. hindsii* | Wang et al.2023 |
| SRR22345206 | *Fortunella* | *F. hindsii* | Wang et al.2023 |
| XTS01 | *XTS* |  | This study |
| XTS02 | *XTS* |  | This study |
| XTS03 | *XTS* |  | This study |
| KBG4 | *Poncirus* | *Poncirus trifoliat* | This study |
| KBG6 | *Poncirus* | *Poncirus trifoliat* | This study |
| EM01 | *papeda* | *Papeda ichangensis* | This study |
| EM03 | *papeda* | *Papeda ichangensis* | This study |
| EM04 | *papeda* | *Papeda ichangensis* | This study |

**Supplementary Table4 Genome-wide pairwise genetic distances (1 − IBS) within and among selected citrus lineages**

| Comparison | Number of pairs | Mean | Median | SD | Range |
| --- | --- | --- | --- | --- | --- |
| Within XTS | 3 | 0.0621 | 0.0629 | 0.0015 | 0.0603–0.0629 |
| Within *Fortunella* | 55 | 0.1235 | 0.1252 | 0.0054 | 0.0989–0.1305 |
| XTS–*Fortunella* | 33 | 0.1476 | 0.1494 | 0.0057 | 0.1341–0.1566 |
| *Fortunella*–*Poncirus* | 22 | 0.1467 | 0.1487 | 0.0086 | 0.1277–0.1586 |
| *Fortunella*–*Papeda* | 33 | 0.1673 | 0.1693 | 0.0064 | 0.1513–0.1746 |

Genetic distance was calculated as 1 − identity by state (IBS) using genome-wide SNPs. “Within XTS” represents pairwise comparisons among the three sampled individuals of the Xiangtoushan lineage. Pairwise distances are presented as descriptive comparisons; because individual samples contribute to multiple pairs, the pairwise observations are not statistically independent.

**Supplementary Table S5. Local quartet support for internal branches of the ASTRAL species tree inferred from 5,865 single-copy nuclear gene trees**

| **Node** | **Clade** | **Local PP** | **q1** | **q2** | **q3** | **QC** | **EN** |
| --- | --- | --- | --- | --- | --- | --- | --- |
| **N11** | *Hongdaicitrus* + *Fortunella* (focal branch) | 1 | 70.55 | 14.1 | 15.36 | 198 | 5865 |
| **N01** | *Citrus medica + Citrus indica* | 1 | 63.55 | 26.03 | 10.42 | 16 | 5865 |
| **N02** | *Citrus medica + Citrus indica + Microcitrus australasica* | 1 | 48.19 | 7.46 | 44.34 | 56 | 5865 |
| **N03** | *Citrus medica + Citrus indica + Microcitrus australasica + Microcitrus australis + Eremocitrus glauca* | 1 | 40.74 | 38.19 | 21.07 | 144 | 5865 |
| **N04** | *Microcitrus australis + Eremocitrus glauca* | 1 | 79.01 | 10.37 | 10.62 | 42 | 5865 |
| **N05** | *Citrus medica + Citrus indica + Microcitrus australasica + Microcitrus australis + Eremocitrus glauca + Papeda ichangensis + Citrus maxima* | 1 | 36.61 | 29.53 | 33.86 | 320 | 5865 |
| **N06** | *Papeda ichangensis + Citrus maxima* | 1 | 61.19 | 16.78 | 22.03 | 60 | 5865 |
| **N07** | *Citrus medica + Citrus indica + Microcitrus australasica + Microcitrus australis + Eremocitrus glauca + Papeda ichangensis + Citrus maxima + Hongdaicitrus xiangtoushanensis + Fortunella hindsii + Fortunella japonica + Citrus reticulata (mangshanyeju) + Citrus tachibana + Citrus ryukyuensis* | 1 | 39.73 | 25.38 | 34.89 | 168 | 5865 |
| **N08** | *Citrus medica + Citrus indica + Microcitrus australasica + Microcitrus australis + Eremocitrus glauca + Papeda ichangensis + Citrus maxima + Hongdaicitrus xiangtoushanensis + Fortunella hindsii + Fortunella japonica + Citrus reticulata (mangshanyeju) + Citrus tachibana + Citrus ryukyuensis + Citrus mangshanensis* | 1 | 49.15 | 28.75 | 22.11 | 30 | 5865 |
| **N09** | *Poncirus polytrifolia + Poncirus trifoliata* | 1 | 89.45 | 7.31 | 3.23 | 16 | 5865 |
| **N10** | *Hongdaicitrus xiangtoushanensis + Fortunella hindsii + Fortunella japonica + Citrus reticulata (mangshanyeju) + Citrus tachibana + Citrus ryukyuensis* | 1 | 38.34 | 32.68 | 28.98 | 420 | 5865 |
| **N12** | *Fortunella hindsii + Fortunella japonica* | 1 | 54.22 | 25.55 | 20.24 | 42 | 5865 |
| **N13** | *Citrus reticulata (mangshanyeju) + Citrus tachibana + Citrus ryukyuensis* | 1 | 82.4 | 9.8 | 7.79 | 110 | 5865 |
| **N14** | *Citrus reticulata (mangshanyeju) + Citrus tachibana* | 1 | 48.75 | 25.99 | 25.25 | 16 | 5865 |

q1, q2, and q3 represent the local quartet frequencies supporting the ASTRAL-inferred topology and the two alternative quartet topologies, respectively. Local PP indicates the local posterior probability of the ASTRAL-inferred topology. QC is the number of quartet sets around each internal branch, and EN is the effective number of gene trees contributing to that branch. Quartet frequencies are presented as percentages.

**Supplementary Table S6. D-statistics testing asymmetric allele sharing among *Hongdaicitrus*, *Fortunella*, *Papeda*, and *Citrus***

| P1 | P2 | P3 | Outgroup | D statistic | SE | Z-score | P-value | BH-adjusted P-value | f4-ratio | BBAA | ABBA | BABA |
| --- | --- | --- | --- | --- | --- | --- | --- | --- | --- | --- | --- | --- |
| *Hongdaicitrus* | *Fortunella* | *Papeda* | *Poncirus* | 0.066071 | 0.007854 | 8.413 | 2.300e-16 | 9.200e-16 | 0.130087 | 8416.9 | 4723.51 | 4138.02 |
| *Hongdaicitrus* | *Fortunella* | *Citrus* | *Poncirus* | 0.009633 | 0.007071 | 1.362 | 1.731e-01 | 1.731e-01 | 0.028783 | 8722.28 | 3625.34 | 3556.16 |
| *Papeda* | *Fortunella* | *Citrus* | *Poncirus* | 0.019717 | 0.007283 | 2.707 | 6.785e-03 | 1.357e-02 | 0.058022 | 5193.59 | 3741.59 | 3596.9 |
| *Papeda* | *Hongdaicitrus* | *Citrus* | *Poncirus* | 0.011668 | 0.007664 | 1.523 | 1.279e-01 | 1.705e-01 | 0.031152 | 4045.71 | 3235.93 | 3161.29 |

D-statistics were calculated from 534,534 biallelic SNPs using *Poncirus* as the outgroup. Z-scores and P-values were estimated using 100 block-jackknife replicates. BH-adjusted P-values were calculated across the four tests. Positive D values indicate excess allele sharing between P2 and P3 relative to P1 and P3.

**Supplementary Table S7. Per-sample gene recovery and missing-data statistics for the 5,873 single-copy nuclear orthologues used in phylogenomic analyses.**

| Sample | Recovered_loci | Total_loci | Gene_recovery_rate_percent | Nonmissing_sites | Missing_sites | Total_sites | Missing_data_percent |
| --- | --- | --- | --- | --- | --- | --- | --- |
| Atalantia_buxfoliata | 5873 | 5873 | 100 | 9128820 | 135621 | 9264441 | 1.4639 |
| Citrus_mangshanensis | 5873 | 5873 | 100 | 9132909 | 131532 | 9264441 | 1.4198 |
| Citrus_mangshanyeju | 5873 | 5873 | 100 | 9262761 | 1680 | 9264441 | 0.0181 |
| Citrus_maxima | 5873 | 5873 | 100 | 8931795 | 332646 | 9264441 | 3.5906 |
| Citrus_medica | 5873 | 5873 | 100 | 9045327 | 219114 | 9264441 | 2.3651 |
| Citrus_trifoliata | 5873 | 5873 | 100 | 9024150 | 240291 | 9264441 | 2.5937 |
| EM04 | 5873 | 5873 | 100 | 9204795 | 59646 | 9264441 | 0.6438 |
| Fortunella_hindsii | 5873 | 5873 | 100 | 9120969 | 143472 | 9264441 | 1.5486 |
| KBG4 | 5873 | 5873 | 100 | 9211464 | 52977 | 9264441 | 0.5718 |
| SRR14482355 | 5873 | 5873 | 100 | 9259485 | 4956 | 9264441 | 0.0535 |
| SRR14766314 | 5873 | 5873 | 100 | 9257808 | 6633 | 9264441 | 0.0716 |
| SRR17027002 | 5873 | 5873 | 100 | 9237900 | 26541 | 9264441 | 0.2865 |
| SRR17035236 | 5872 | 5873 | 99.983 | 9179718 | 84723 | 9264441 | 0.9145 |
| SRR25399167 | 5865 | 5873 | 99.8638 | 7183248 | 2081193 | 9264441 | 22.4643 |
| SRR3948493 | 5873 | 5873 | 100 | 9193242 | 71199 | 9264441 | 0.7685 |
| SRR6188449 | 5873 | 5873 | 100 | 9256068 | 8373 | 9264441 | 0.0904 |
| XTS01 | 5873 | 5873 | 100 | 9240024 | 24417 | 9264441 | 0.2636 |
| XTS02 | 5873 | 5873 | 100 | 9250278 | 14163 | 9264441 | 0.1529 |
| XTS03 | 5873 | 5873 | 100 | 9244395 | 20046 | 9264441 | 0.2164 |

A locus was considered recovered when the corresponding sequence contained at least one unambiguous nucleotide. Gaps, Ns, and other ambiguous character states were treated as missing data.
